# Linking the Evolution of Raman-Active Vibrational Modes to the Glass Transition of a Polymer Mixed Conductor

**DOI:** 10.1021/acs.chemmater.6c01379

**Published:** 2026-08-29

**Authors:** Giannis G. Gkikas, Elsa Veronica Flores-Vela, Mariavittoria Craighero, Joost Kimpel, Christian Müller, Anna Martinelli

**Affiliations:** Department of Chemistry and Chemical Engineering, 11248 Chalmers University of Technology, Gothenburg 41296, Sweden

## Abstract

Thin films and bulk samples of an organic mixed ionic-electronic conductor (OMIEC), i.e., a thienothiophene (TT) based conjugated polymer with oligoether side chains, are investigated using variable-frequency dynamic mechanical thermal analysis (DMTA) and variable-temperature Raman spectroscopy. Macroscopically observed thermal transitions are correlated with molecular-scale dynamics. This multianalytical approach identifies a low-temperature β-relaxation originating from side-chain motion and a higher-temperature α-relaxation linked to backbone rearrangement. Specifically, temperature-dependent Raman spectra reveal shifts in characteristic C=C/C–C, C–H, and C–S–C vibrational modes, which were quantified using a sigmoid model. Furthermore, a refined assignment of key vibrational modes ensures accurate spectroscopic interpretation. These results establish temperature-dependent Raman spectroscopy as a sensitive probe for glass and subglass transitions, providing a direct link between vibrational dynamics and thermo-mechanical properties of an exemplar mixed ionic-electronic conductor.

## Graphical abstract

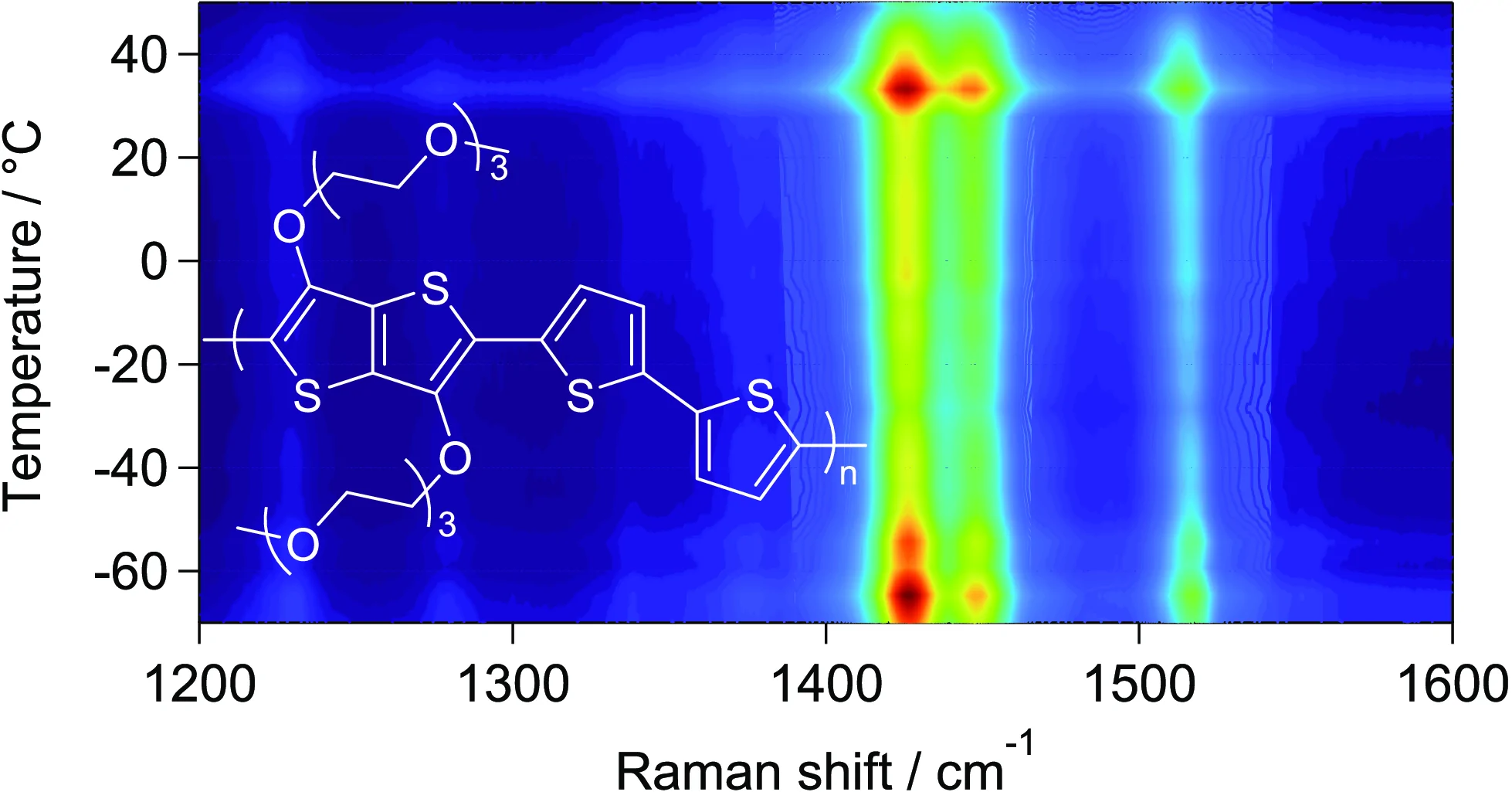

## 1 Introduction

Organic mixed ionic-electronic conductors (OMIECs) based on conjugated polymers receive considerable attention for diverse applications, ranging from wearable electronics and bioelectronics to energy harvesting and storage.^1–3^ Among p-type OMIECs, polymers based on thienothiophene (TT) and bithiophene (T2) have emerged as a prominent class, featuring a conjugated backbone and oligoether side chains that facilitate the transport of electronic and ionic charges, respectively.

For instance, the polymer p­(g_3_T2-TT), bearing triethylene glycol side chains attached to its thiophene units (see Figure S1 for the chemical structure), exhibits an electronic charge-carrier mobility exceeding 1 cm^2^ V^–1^ s^–1^ when employed as the channel material of organic electrochemical transistors (OECTs).^4^ Further, the isomeric variant with the triethylene glycol side chains placed on the 3,3′-position of the T2, has mobility values of 5–8 cm^2^ V^–1^ s^–1^, depending on the density of homocoupling defects.^5^ Likewise, for the variant p­(g_3_TT-T2), with the oligoether side chains attached to the thienothiophene units instead (see Figure 1 for chemical structure), mobility values as high as 6 cm^2^ V^–1^ s^–1^ have been reported.^6^ While the electrical and electrochemical properties of OMIECs are increasingly well understood, their thermo-mechanical behavior remains less explored. This is despite its crucial importance for flexible, wearable, and implantable devices, which can experience temperature variation and mechanical deformation during operation.^7^

**Figure 1:**
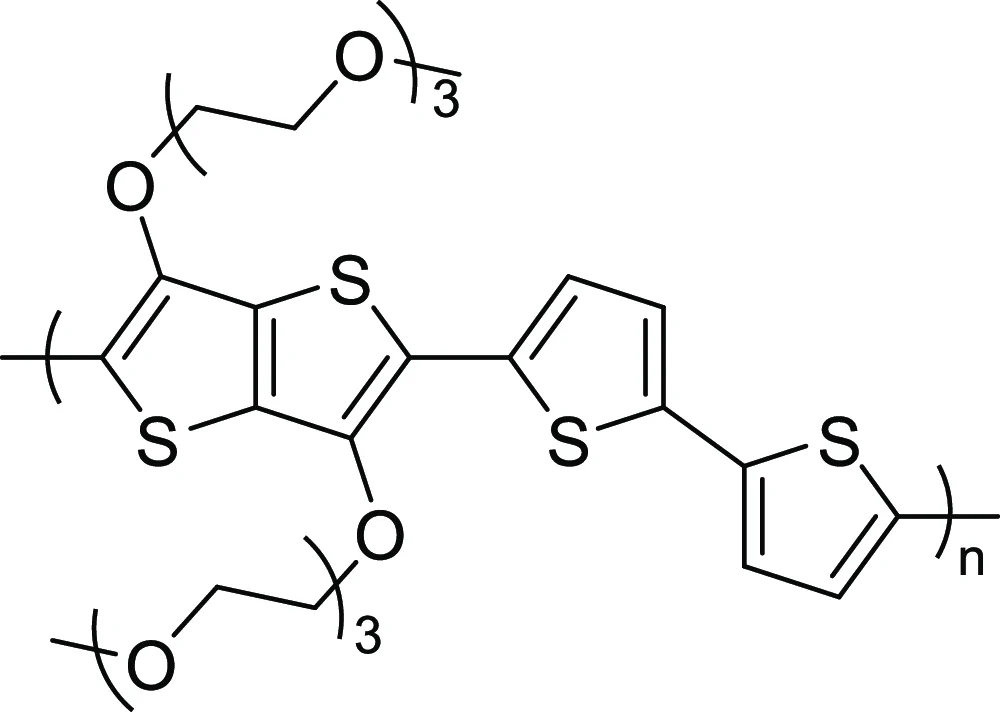
Molecular structure of the conjugated polymer p­(g_3_TT-T2).

Thienothiophene-based polymers typically comprise both ordered and disordered domains, the latter being primarily responsible for ion ingression and transport.^8^ The presence of oligoether side chains contributes to lowering the glass transition temperature *T*_g_ (often being below 0 °C), resulting in high ionic mobility at ambient conditions. Recently, Ding et al.^9^ employed alternating-current (AC) chip calorimetry to study thin films of p­(g_3_T2-TT) and observed two thermal transitions, at −87 and −26 °C (using a frequency of 10 Hz and a heating rate of 1 °C min^–1^). The authors assigned the lower-temperature transition to the relaxation of triethylene glycol side chains, occurring in a temperature range where other materials with oligoether segments also feature a relaxation process.^10,11^ The higher-temperature transition was instead explained with the onset of a backbone relaxation, based on a comparison with a polythiophene with triethylene glycol side chains, whose more flexible backbone results in a lower *T*_g_ compared to p­(g_3_T2-TT).^12^ Interestingly, some of us have observed slightly higher transition temperatures for p­(g_3_TT-T2),^13^ likely reflecting both its higher number-average molecular weight (*M*_*n*_ = 13 vs 6 kg mol^–1^) and a difference in side-chain positioning, which can be expected to modify the S···O interactions along the backbone.

Distinct side- and main-chain relaxation processes have been reported for many types of conjugated polymers. For instance, broadband dielectric spectroscopy of a polyfluorene and various poly­(*p*-phenylene)­s revealed two relaxation processes, a β-relaxation assigned to the onset of side-chain motion and an α-relaxation due to backbone movement.^14–16^ Likewise, dynamic mechanical thermal analysis (DMTA) has been widely used, revealing two, and sometimes three, relaxation processes in the case of poly­(3-alkylthiophene)­s and donor–acceptor copolymers based on e.g., quinoxaline, naphthalenetetracarboxylic diimide, or benzodithiophene.^17–22^ However, the microscopic origin of these transitions is not disclosed by these techniques and is instead often inferred by either comparing with results from dynamic scanning calorimetry, which typically only reveals the *T*_g_, or by analyzing the frequency dependence of the underlying relaxation processes.^23,24^

By contrast, Raman spectroscopy is a powerful and noninvasive experimental method that can probe structural and conformational changes microscopically, i.e., at a molecular level. For classical polymers such as polystyrene and polyamide, Raman spectroscopy has been used to detect glass transitions and local relaxations.^25,26^ However, in the field of conjugated polymers, Raman spectroscopy has mainly served as a tool to probe structural identity, doped states, and crystallization phenomena,^27,28^ but to the best of our knowledge, it has not been implemented to investigate and correlate molecular relaxations.

We here fill this knowledge gap, demonstrating that, if appropriately employed, temperature-dependent Raman spectroscopy can indeed probe the glass and subglass transitions macroscopically observed in conjugated polymer films, enabling a direct connection to their origin on a molecular level.

In order to use this method, we carefully assigned key vibrational modes in p­(g_3_TT-T2), which has not been studied before by Raman spectroscopy. This polymer shares backbone and molecular units with polymers like poly­[2,5-bis­(3-tetradecylthiophen-2-yl)­thieno­[3,2-*b*]­thiophene] (PBTTT) and p­(g_3_T2-TT), hence the assignment proposed here is relevant for a wide range of materials. Specifically, we clarify that C=C stretching modes in different aromatic units (thienothiophene and thiophene) can be spectroscopically distinguished, and we provide a consistent assignment of C–C bonds within and in-between the aromatic rings.^29–31^ Since the vibrational coupling in thienothiophene-based backbones is notably complex,^27^ a precise assignment was essential to correctly link specific vibrational modes to structural relaxation processes.

In this work, we combine variable-frequency DMTA with variable-temperature Raman spectroscopy to unambiguously relate the thermal transitions observed macroscopically for p­(g_3_TT-T2) films to local dynamic events of specific molecular groups. By comparing the experimentally recorded Raman spectrum of the polymer with the spectra of its structural building blocks (i.e., thienothiophene and bithiophene) and of other related thienothiophene-based polymers (i.e., PBTTT, p­(g_3_T2-TT), and the copolymer p­(g_3_TT-T) comprising thienothiophene and a single thiophene unit, see Figure S1 for the chemical structure of these reference compounds), we were able to assign key spectral features. The temperature dependence of these vibrational modes is then correlated to relaxation processes revealed by DMTA. This approach allows us to indisputably assign the two observed thermal transitions to the onset of side- and main-chain relaxations, respectively. Ultimately, we establish Raman spectroscopy as a valuable tool for capturing glass and subglass transition processes in conjugated polymer films.

## 2 Experimental Section

### 2.1 Materials

Thiophene (99%), thieno­[3,2-*b*]­thiophene (95%), and 2,2′-bithiophene (97%), as well as triethylene glycol monomethyl ether (97%) and tetraethylene glycol dimethyl ether (98%), were purchased from Sigma–Aldrich. Chloroform (99%) was purchased from Fisher Scientific. Poly­(3-hexylthiophene) (P3HT) with a regioregularity of 94% was purchased from Ossila, with a number-average molecular weight *M*_*n*_ = 13 kg mol^–1^ and a dispersity *Đ* = 2.7. All chemicals were used as received without further purification. The 3,6-bis­(triethylene glycol monomethyl ether)­thieno­[3,2-*b*]­thiophene (g_3_TT) monomer, poly­(3,6-bis­(2-methoxyethoxy)-2-methyl-5-(5′-methyl-[2,2′-bithiophen]-5-yl)­thieno­[3,2-*b*]­thiophene), p­(g_3_TT-T2), with *M*_*n*,NMR_ = 9 kg mol^–1^, *M*_*n*,SEC_ = 13 kg mol^–1^, and *Đ* = 1.5, and poly­(3,6-bis­(2-methoxyethoxy)-2-methyl-5-(5-methylthiophen-2-yl)­thieno­[3,2-*b*]­thiophene), p­(g_3_TT-T), with *M*_*n*,NMR_ = 14 kg mol^–1^, *M*_*n*,SEC_ = 14 kg mol^–1^, and *Đ* = 6.4, were synthesized as reported previously.^6,32^

### 2.2 Thin Film Preparation

For the Raman experiments, thiophene, thieno­[3,2-*b*]­thiophene, 2,2′-bithiophene, and P3HT were measured as solids. Solutions of p­(g_3_TT-T2) and p­(g_3_TT-T) were prepared at a concentration of 8 g L^–1^ in chloroform. From these solutions, a volume of 75 μL was used for spin-coating thin films (approximately 50–150 nm thick) onto Si substrates that had been precleaned by sonication in acetone, followed by isopropanol. Before polymer deposition, the substrates were treated with toluene, which was spin-coated at a speed of 3000 rpm and an acceleration of 1000 rpm s^–1^ for 60 s. The polymer films were spin-coated at a speed of 1000 rpm and an acceleration of 500 rpm s^–1^ for 60 s. After spin coating, the polymer films were annealed at 80 °C for 15 min. The dried spin-coated films were immediately transferred to the Raman spectrometer for investigation (Figure S2), minimizing oxidation of the polymer, which may occur due to its low ionization energy.^32^

### 2.3 Thermogravimetric Analysis (TGA)

Thermogravimetric analysis (TGA) was conducted using a Mettler Toledo TGA/DSC 3+ instrument under a nitrogen atmosphere. A 2.9 mg sample of p­(g_3_TT-T2) was weighed into an alumina crucible and heated from 30 to 600 °C at a rate of 10 °C min^–1^.

### 2.4 Dynamic Mechanical Thermal Analysis

A free-standing film of p­(g_3_TT-T2), with a thickness of 0.2 mm (length of 7 mm and width of 2 mm), was prepared by compression molding between aluminum foil using a LabPro200 press from Fontijne at 180 °C. TGA measurements confirmed the thermal stability of the material at processing temperatures, with thermal degradation onset at 312 °C (Figure S3). Multifrequency DMTA was performed in tensile mode using a Discovery 850 dynamic mechanical analyzer from TA Instruments. The sample was mounted with a preload force of 5 mN, and a 0.02% oscillatory strain was applied at temperatures ranging from −80 to 38 °C. The frequency was varied from 0.1 to 25 Hz, while the temperature was increased in steps of 2.5 °C with a waiting time of 5 min after each increment. The peaks in tan δ were used to estimate transition temperatures.

### 2.5 Fast Scanning Calorimetry (FSC)

A p­(g_3_TT-T2) solution of 4 g L^–1^ in chloroform was drop cast onto a UFS 2 chip sensor from Mettler Toledo. Measurements were performed using a Mettler Toledo Flash DSC 1. The sample was repeatedly heated to 150 °C at a rate of 2000 K s^–1^ and then cooled to −80 °C at rates ranging from 0.1 to 1000 K s^–1^. The solution concentration was optimized to obtain a sufficiently thin film (≤100 nm) for reliable Flash DSC measurements at heating and cooling rates up to 2000 K s^–1^ while maintaining adequate signal intensity.

### 2.6 Raman Spectroscopy

To collect room temperature and temperature-dependent Raman spectra, an InVia Reflex confocal Raman spectrometer from Renishaw was used that, by use of the 532 nm laser as the excitation and the 2400 l mm^–1^ grating, could provide a spectral resolution better than 1 cm^–1^. For the collection of the backscattered light, a Peltier-cooled CCD array detector was used, which ensured low noise and high thermal stability during spectral acquisition. Before each experiment, calibration was performed using a Si wafer, referencing the strong first-order transverse optical phonon mode to 520.6 cm^–1^.

For the purpose of peak assignment, an Ar-ion laser delivering at 532 nm was used, at a power carefully limited to between 0.1 and 5.0 mW to avoid thermal damage. To focus the laser on the sample, a 50× Leica objective (NA = 0.50) was used. For each sample, three different spectra were collected at three distinct spots. For each measurement, 4 accumulations with 10 s of exposure time were collected, in the extended mode, scanning the spectral range from 100 to 4000 cm^–1^.

To record variable-temperature Raman spectra, a Linkam THMSG 600 heating–cooling stage cooled with liquid N_2_ was used. The temperature was scanned from 65 to −70 °C and back, using a cooling/heating rate of 20 °C min^–1^. The Linkam cell was controlled using the WiRE 3.3 software provided by Renishaw. The 532 nm laser source set at 5 mW of power was used, in combination with a 50× Leica objective. The Raman spectra were collected in static mode, covering the fixed spectral window of 600–1700 cm^–1^ in one single experiment, then covering 2500–3400 cm^–1^ in another independent experiment run with identical optical parameters. Each spectrum was acquired with one accumulation of 1 s, the static mode ensuring a shorter exposure time of the sample to the laser. This matches the rapid cooling/heating rates needed to observe glass transitions, without compromising Raman intensity.

For further analysis of Raman shift changes, all Raman spectra were reduced to zero intensity at 611 cm^–1^ (applying a constant subtraction) and then normalized to the intensity of the vibrational mode with peak at 1515 cm^–1^. This is an arbitrary choice that does not affect the estimation of the Raman shifts. A Multipeak fitting procedure provided by the software Igor Pro 9.05 was then applied to all spectra using a linear baseline and Lorentzian functions, treating the subregions 2710–3104, 1300–1600, 1260–1299, 1200–1260, 1000–1101, and 720–780 cm^–1^ separately. Peak positions were extracted from this peak fitting procedure and plotted as a function of temperature for further analysis and interpretation. The details of the fitting procedure adopted for these spectral regions are shown in Figure S4, for spectra recorded at room temperature as a representative case.

## 3 Results and Discussion

### 3.1 Mechanical Analysis and Thermal Transitions

DMTA was performed at frequencies, *f*, ranging from 0.1 to 25 Hz, to determine the thermal transitions that the polymer p­(g_3_TT-T2) experiences (Figure 2). Two relaxation processes can be distinguished, as evidenced by the two distinct peaks in the loss tangent, tan δ, curve. That is, a low-temperature relaxation in the range from −60 to −45 °C and a higher-temperature relaxation in the range from −30 to 10 °C. The lower-temperature process occurs at temperatures close to the glass transition temperature of poly­(ethylene oxide), reported to be *T*_g_ ≃ −50 °C.^33^ The observed process is therefore assigned to the relaxation of the oligoether side chains, hence being a subglass-transition relaxation occurring at *T*_β_. The activation energy, *E*_a_, of the β-relaxation was determined by describing the temperature dependence of the tan δ peak position with an Arrhenius equation, according to

1 $$f=f_{0}e^{-E_{a}/\left(\mathrm{RT}\right)}$$

where *f*_0_ is a pre-exponential frequency factor, *R* is the ideal gas constant, and *T* is the absolute temperature in Kelvin. The β-relaxation typically follows an Arrhenius dependence and is therefore less temperature-sensitive than the strongly non-Arrhenius α-relaxation.^34^ The linear fit to the data shown in the Arrhenius plot (Figure 2b) yields *E*_a_ = (229 ± 18) kJ mol^–1^, i.e., (2.37 ± 0.20) eV.

**Figure 2:**
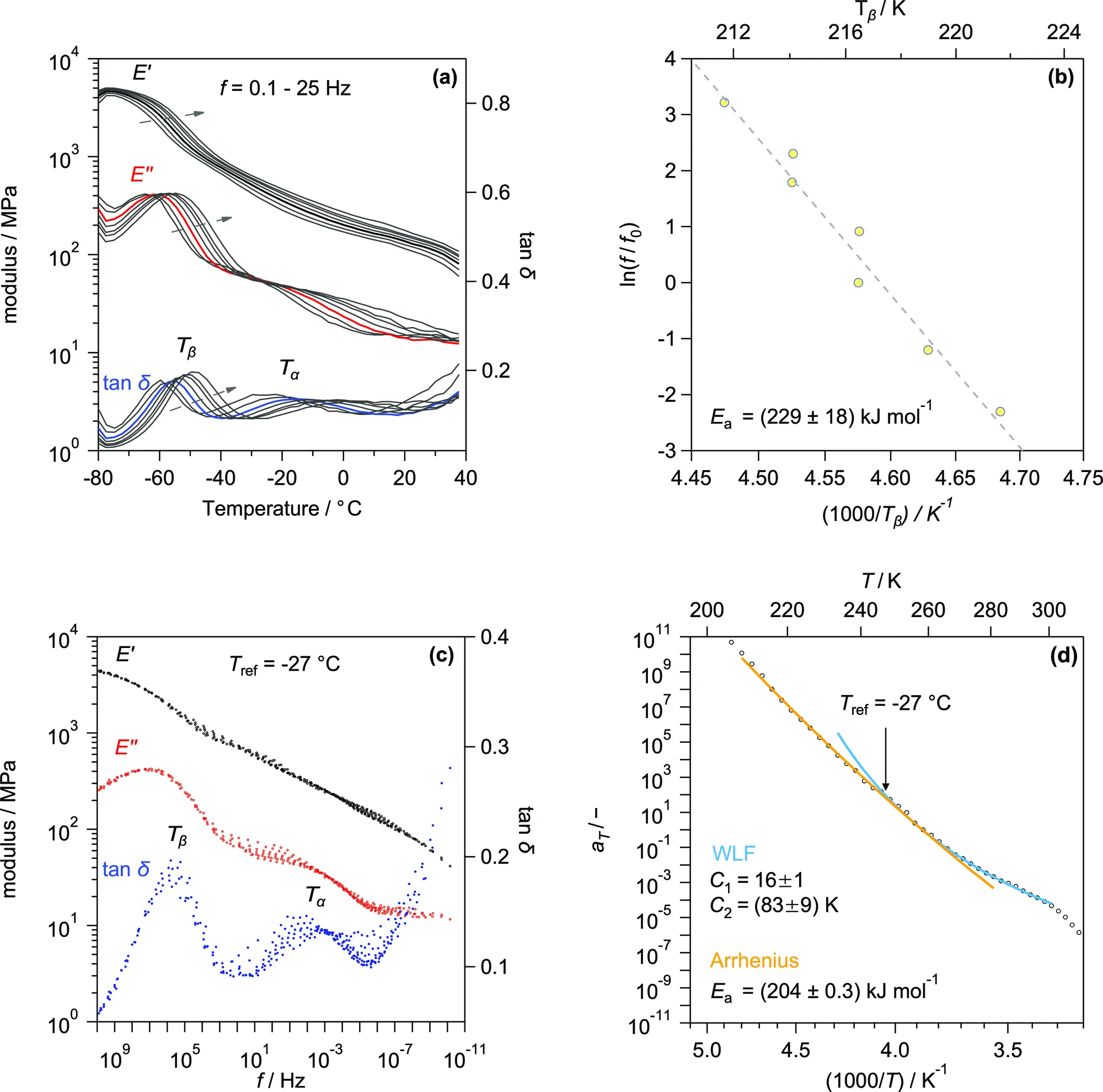
(a) Storage and loss moduli (*E*′ and *E*″) and loss tangent (tan δ = *E*″/*E*′) recorded by DMTA at frequencies ranging from *f* = 0.1 to 25 Hz (see direction of arrows; bold lines correspond to 1 Hz); (b) Arrhenius plot and linear fit (dashed line) of tan δ peak temperatures *T*_β_ associated with the β-relaxation process; (c) master curves for *E*′, *E*″, and tan δ at *T*_ref_ = −27 °C; and (d) DMTA frequency shift factors (*a*_*T*_) such that *E*′(*f*, *T*) = *E*′(*f*_α_, *T*_ref_), together with Arrhenius and Williams–Landel–Ferry (WLF) fits for *T* < *T*_ref_ and *T* > *T*_ref_, respectively.

We hypothesize that the higher-temperature process corresponds to the relaxation of the backbone of p­(g_3_TT-T2), occurring at a temperature that we refer to as *T*_α_. A master curve in the frequency domain was constructed and the reference temperature *T*_ref_ was selected so that the peak in the loss tangent, tan δ, associated with the α-relaxation was located at *f*_α_ = 10^–2^ s^–1^, i.e., the relaxation time τ_α_ = *f*_α_^–1^ = 100 s (Figure 2c), which is a characteristic value at *T*_g_.^35^ Interestingly, the polymer is stiff with a storage modulus *E*′ > 1 GPa at frequencies *f* > 10^8^ Hz, i.e., above the frequency range where the β-relaxation can take place, and then drops to below 0.3 GPa at *f*_α_, where the backbone can relax. The shift factor *a*_*T*_ obtained from the construction of the master curve using *T*_ref_ = −27 °C can be described with the Williams–Landel–Ferry (WLF) model for temperatures *T* > *T*_ref_

2 $$\log\left(a_{T}\right)=-\frac{C_{1}\left(T-T_{\mathrm{ref}}\right)}{C_{2}+\left(T-T_{\mathrm{ref}}\right)}$$

where *C*_1_ and *C*_2_ are fitting parameters. A fit of *a*_*T*_ in a temperature window around *T*_ref_ = −27 °C yielded values of *C*_1_ = 16 ± 1 and *C*_2_ = (83 ± 9) K (Figure 2d), which are close to values typically reported for flexible-chain commodity polymers.^36^ For *T* < *T*_ref_, an Arrhenius equation can be used to describe the relaxation time, and thus, *a*_*T*_ can be described as

3 $$\log\left(a_{T}\right)=\frac{E_{a}}{R\ln\left(10\right)}\left(\frac{1}{T}-\frac{1}{T_{\mathrm{ref}}}\right)$$

A fit of *a*_*T*_(*T*) across temperatures below *T*_ref_ = −27 °C yielded *E*_a_ = (204 ± 0.3) kJ mol^–1^, i.e., (2.114 ± 0.003) eV (Figure 2d). Our analysis indicates that Arrhenius and WLF type behavior can describe *a*_*T*_(*T*) below and above *T*_ref_ = −27 °C, respectively, as expected for the *T*_g_ of polymeric materials.

DMTA was also performed at variable scan rates of 3, 5, and 15 °C min^–1^ (Figure S5). The DMTA thermograms reveal that the two dynamic transitions consistently appear, independent of the scan rate used. In addition, to overcome the low sensitivity of conventional DSC toward glass transitions, we employed fast scanning calorimetry (FSC). FSC thermograms revealed a clear relaxation at −40 °C (Figure S6), which is consistent with the β-relaxation observed by DMTA (Figure 2a). The higher-temperature relaxation observed by DMTA was, however, not visible by FSC, motivating the use of vibrational spectroscopy to better understand its origin and nature. Furthermore, the fragility was calculated to be *m* = 41 ± 1 (from the slope at values close to *T*_ref_, Figure 2d), indicating that p­(g_3_TT-T2) is a relatively strong glass former compared to many conventional polymers.^37^

### 3.2 Refined Assignment of Vibrational Modes

Raman spectra of p­(g_3_TT-T2) were recorded at room temperature using the 532 nm laser as the excitation source (Figure 3). Although the polymer was measured as a thin film (50–150 nm in thickness), the Raman intensity is remarkably high. This is attributed to the large scattering cross section of the aromatic rings combined with resonance effects, as the polymer exhibits an absorption maximum around 600 nm. The Raman spectrum of this polymer has not been reported before; thus, we aimed for a detailed assignment of its main vibrational modes. Accordingly, we analyzed the Raman spectra of its building blocks and how they change upon small structural modifications, while we also made comparisons to spectral trends already reported in the literature for similar polymers.

**Figure 3:**
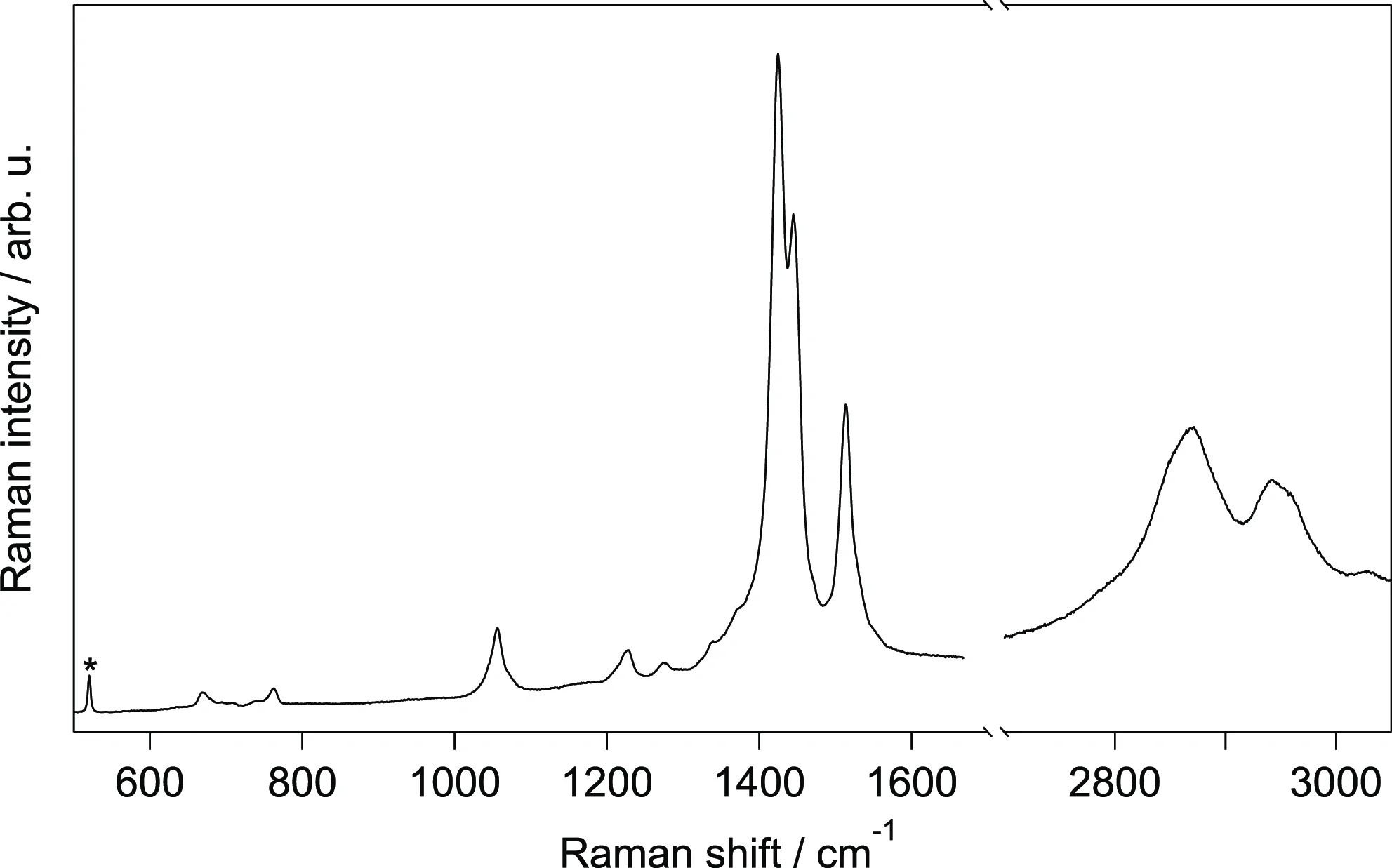
Raman spectrum of a thin film of p­(g_3_TT-T2) recorded at room temperature. The asterisk marks the vibration at 520.6 cm^–1^ arising from the Si substrate onto which the thin film was deposited.

In the high-frequency range of 2700–3100 cm^–1^, two main features are observed, which we assign to the C–H stretching modes in the side chains of p­(g_3_TT-T2). Deconvolution of this spectral range (Figure S4a) and direct comparison with the Raman spectra independently recorded for triethylene glycol monomethyl ether and tetraethylene glycol dimethyl ether (Figure S7), suggest that the low-frequency feature includes symmetric C–H stretching in CH_2_ and CH_3_ units. The lower-intensity feature observed at higher frequencies includes antisymmetric C–H stretching in CH_2_ and CH_3_ units.^38^ In the analysis of the temperature-dependent Raman spectra that will follow, focus will be on the main feature at 2860 cm^–1^ only, its deconvolution being unambiguous and the assignment reliable.

In the 1200–1600 cm^–1^ region, the characteristic, strong signatures of conjugated polymers appear, which primarily include C–C and C=C stretching modes in the backbone, consistent with the effective conjugation coordinate (ECC) theorem.^39^ More specifically, peak fitting revealed six contributions between 1300 and 1600 cm^–1^ (Figure S4b), the three most intense modes in p­(g_3_TT-T2) being at 1425, 1447, and 1515 cm^–1^ (Figure 4a). Shoulders to these bands and additional weaker peaks are also resolved. To enable a precise assignment, Raman spectra were also recorded for the crystals of individual building blocks of p­(g_3_TT-T2), i.e., TT (Figure 4b) and T2 (Figure 4c), while Raman spectra of their oligomeric and polymeric forms have been taken from the literature.^40–42^

**Figure 4:**
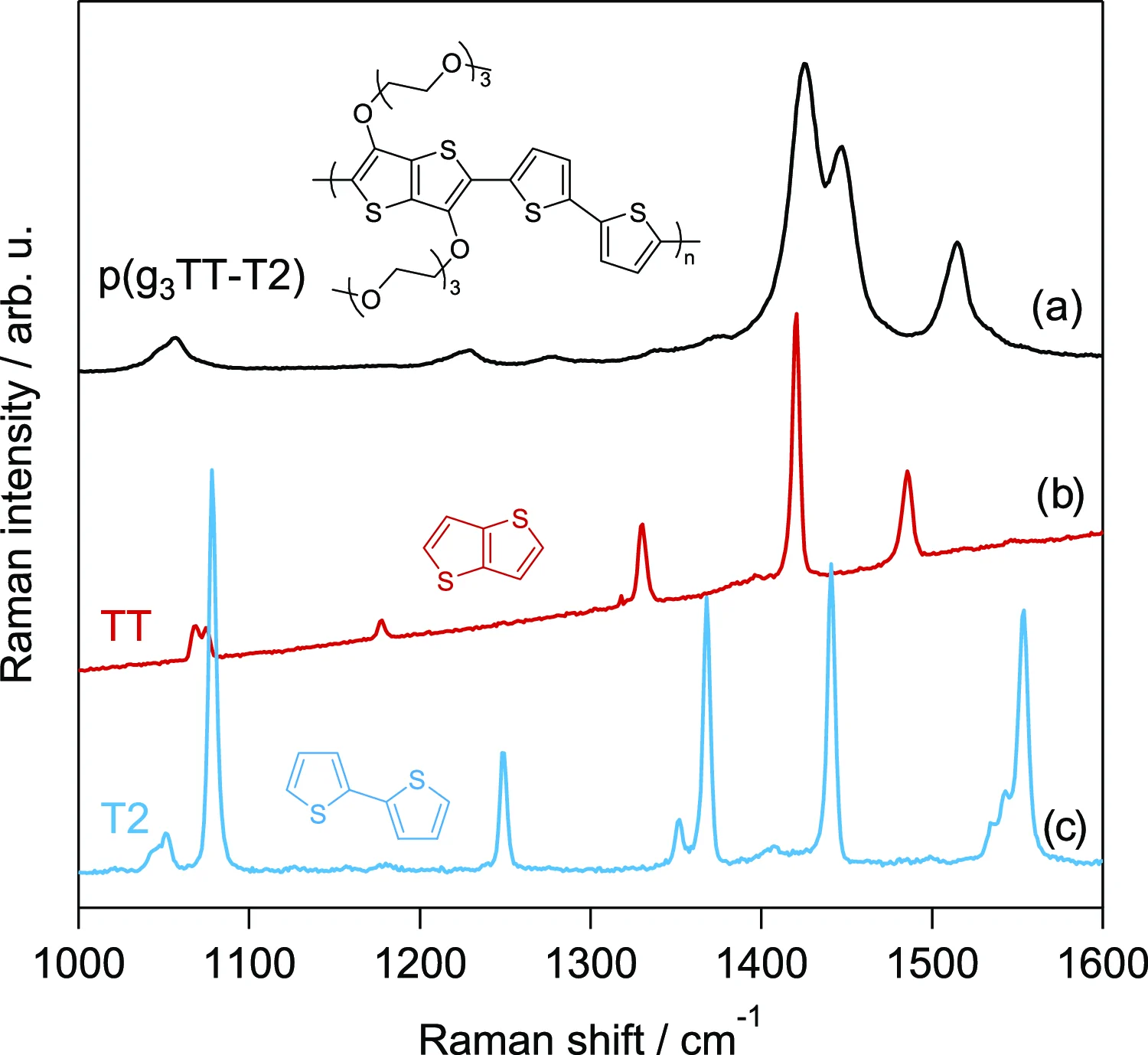
Raman spectra recorded at room temperature in the −C–C– and –C=C– stretching region for (a) a thin film of p­(g_3_TT-T2), (b) thieno­[3,2-*b*]­thiophene (TT), and (c) 2,2′-bithiophene (T2), the latter two as crystals. The molecular structure of each compound is also provided.

In the Raman spectrum of the T2 unit, the strongest modes are found at 1441, 1554, and ∼1370 cm^–1^ (Figure 4c). From spectra already reported in the literature, we infer that upon polymerization, the main mode at 1441 cm^–1^ changes only marginally in wavenumber and does not vary in intensity, whereas the one at 1554 cm^–1^ undergoes a red shift and loses intensity. In contrast, upon polymerization, the mode at about 1370 cm^–1^ loses intensity without shifting.

Based on these observations, but in particular thanks to the insightful work by Brambilla et al.,^43^ who used selective deuteration to distinguish and appropriately assign different vibrations in P3HT, the modes observed in the Raman spectrum of p­(g_3_TT-T2) are assigned as follows: the one at 1447 cm^–1^ to −C=C– symmetric stretching modes of the thiophene backbone, the one at 1515 cm^–1^ to antisymmetric −C=C– stretching of the thiophene backbone, and the one at 1374 cm^–1^ to a vibrational mode that couples stretching of both single and double carbon bonds within the ring units. In addition, the weak vibrational mode observed at ∼1230 cm^–1^ in p­(g_3_TT-T2) is assigned to inter-ring C–C stretching. The corresponding mode observed at ∼1250 cm^–1^ in the T2 spectrum (Figure 4c), but absent in the case of TT, supports this assignment. This mode, however, tends to be extremely weak, as evidenced by the Raman spectra of P3HT.^44^

In the Raman spectra of p­(g_3_TT-T2), a mode of medium intensity can be observed at ∼1050 cm^–1^ (a strong peak at 1080 cm^–1^ in the T2 dimer), which is instead absent in the P3HT spectrum. This is consistent with its attribution to in-plane C–H bending modes, whose intensity persists upon polymerization.

Our peak fitting analysis also reveals a weak contribution at 1476 cm^–1^, which we attribute to CH_2_ and CH_3_ deformation modes of the side chains (Figure S8). These deformation modes were also discernible in the Raman and infrared spectra of P3HT,^43^ for which deuteration enabled the distinction between the modes arising from the hexyl side chains and the modes arising from the backbone of the conjugated polymer, coincidentally falling within the same frequency range. An additional important note is that the main vibration at ∼1445 cm^–1^ is observed at practically the same position in oligothiophenes and P3HT,^40,44^ revealing that the symmetric −C=C– stretching modes are not significantly affected by pendant alkyl side chains.

The intrinsic signatures of the TT unit are also distinct and strong, although systematically red-shifted with respect to the case of the T2 unit (compare Figure 4b and 4c). In the Raman spectrum of TT, the main vibrational modes are found at 1420, 1485, and 1330 cm^–1^ (Figure 4b). Similar to the case of T2 units, also for the TT units, the literature shows that the main vibrational mode assigned to −C=C– symmetric backbone stretching at 1420 cm^–1^ does not shift significantly upon polymerization, whereas the medium intensity mode at 1485 cm^–1^ red shifts and loses intensity, while the mode at 1330 cm^–1^ becomes weaker.^41^ Hence, based on this information, the Raman peak observed at 1425 cm^–1^ in the case of p­(g_3_TT-T2) is assigned to the symmetric −C=C– stretching mode of the TT unit in the polymer.

Likewise, the spectrum of the polymer p­(g_3_TT-T2) contains the distinct features of both units, TT and T2, with some broadening and with the peak assigned to antisymmetric stretching appearing relatively intense and at a frequency between those of each unit, i.e., at 1515 cm^–1^. The presence of the peak at 1050 cm^–1^ in the spectrum of p­(g_3_TT-T2) is rationalized by the triethylene glycol chains being anchored to the TT units, hence not affecting the in-plane (ring) C–H bending modes intrinsic to the T2 units.

The conclusion that the peak at 1445 cm^–1^ in p­(g_3_TT-T2) can be specifically assigned to vibrations in the T2 unit is confirmed by comparing the Raman spectrum of the polymer p­(g_3_TT-T) with that of a p­(g_3_TT-T2), which has a single thiophene rather than a bithiophene as a comonomer to g_3_TT. The only significant difference is the higher intensity of the mode at 1445 cm^–1^ in the case of p­(g_3_TT-T2) (see Figures 5 and S9).

**Figure 5:**
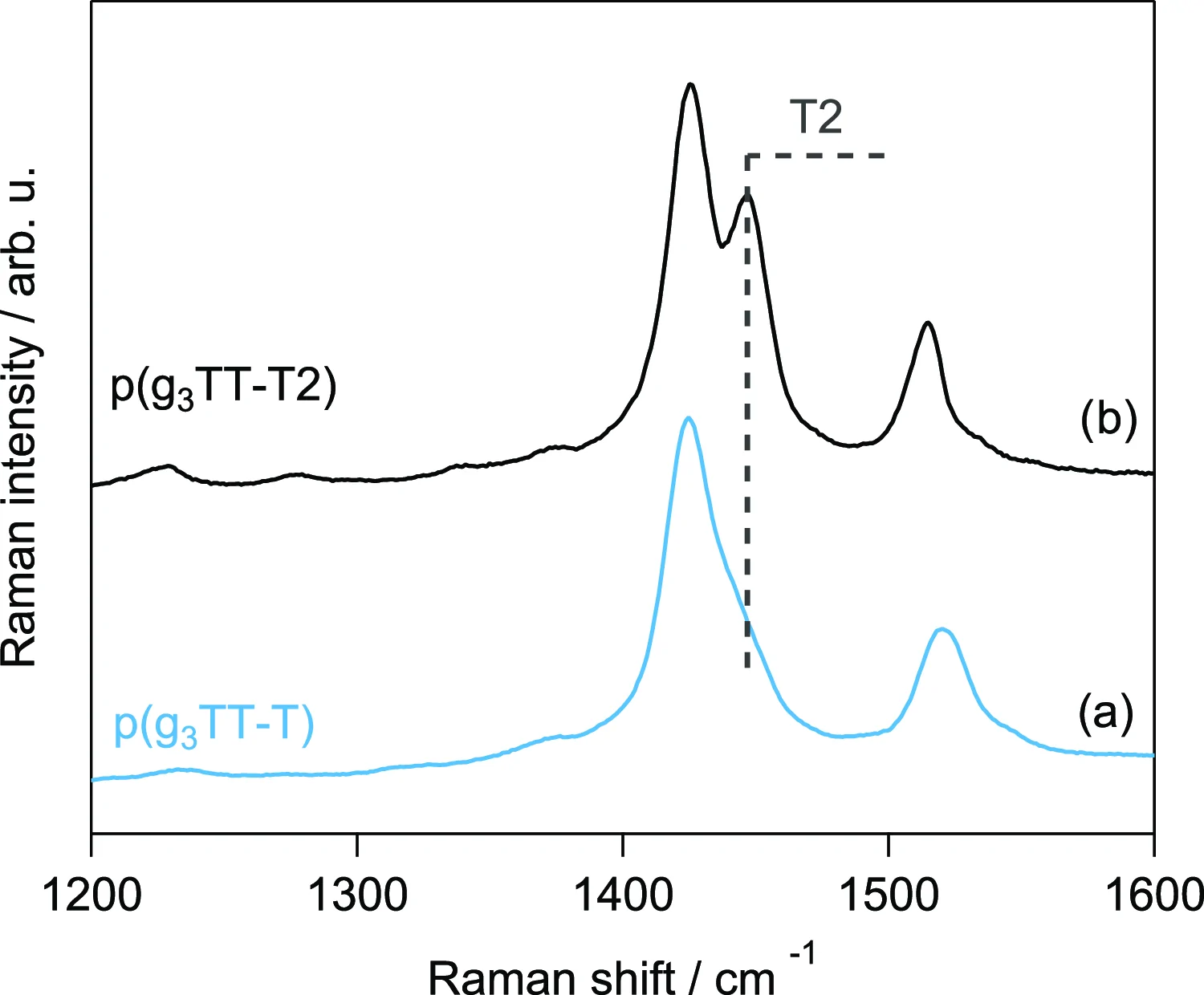
Raman spectra recorded at room temperature from thin films of (a) p­(g_3_TT-T) and (b) p­(g_3_TT-T2).

Further, in the lower-frequency region of 600–800 cm^–1^, a weak double signature is observed, with one feature peaking at 670 cm^–1^ and another peaking at 760 cm^–1^ (Figure 6a). These features are attributed to vibrations that include the C–S–C bending mode,^45^ which, in the Raman spectrum of p­(g_3_TT-T2), appear as two distinct contributions from the TT and T2 units, respectively.

**Figure 6:**
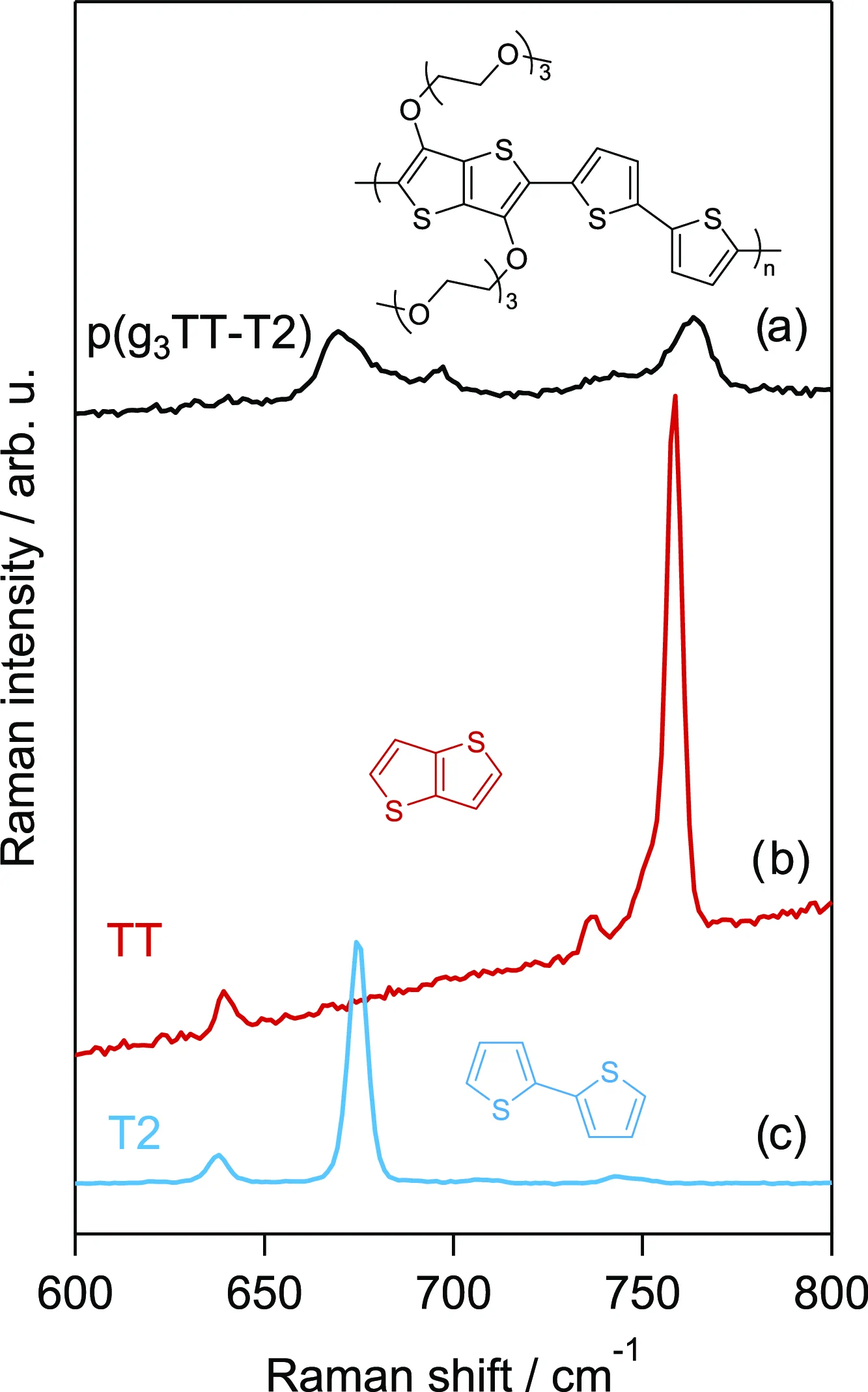
Raman spectra in the δ C–S–C region recorded at room temperature from (a) a p­(g_3_TT-T2) thin film, (b) TT, and (c) T2.

For clarity, the main vibrational modes observed in the Raman spectrum of p­(g_3_TT-T2) are listed in Table 1, along with their assignments and experimentally observed Raman intensities.

**Table 1:** Vibrational Modes Observed in the Raman Spectrum of p­(g_3_TT-T2) and Their Assignment (ν_s_, Symmetric Stretching; ν_as_, Antisymmetric Stretching; δ, Deformation Mode; i.p., In Plane; s, Strong; m, Medium; and w, Weak)

Raman shift (cm^–1^)	vibration	peak intensity
2800–3000	ν_s_ C–H (side chain)	m
1500–1520	ν_as_ –C=C–	s
1430–1450	ν_s_ –C=C– (T2)	s
1400–1430	ν_s_ –C=C– (TT)	s
1300–1390	ν C–C/C=C (ring)	w
1200–1300	ν C–C (inter-ring)	w
1000–1100	δ_ip_ C–H (T2)	m/w
600–800	δ C–S–C	w

### 3.3 Temperature Dependence of Raman Shifts

Having carried out the peak assignment (Table 1), a thin film of p­(g_3_TT-T2) was investigated by variable-temperature Raman spectroscopy, using experimental parameters that enable short collection times (a few seconds), fast cooling/heating scans (to compare with the DMTA analysis), and strong Raman intensities (for reliable peak fitting).

The evolution of the Raman spectrum of p­(g_3_TT-T2) as a function of temperature, covering the range from −70 to 65 °C upon heating, was studied. It is observed that no peaks appear, disappear, or split (Figure 7); however, peak intensity maxima at specific temperatures are clearly observed (Figure S10). These appear as a result of a transient change in the Raman scattering cross section, which can occur in conjunction with relaxation phenomena. For the archetypal polyamide-6,6, for instance, temperature-dependent Raman spectra have revealed clear local intensity maxima at temperatures that correspond to its known α, β, and γ relaxations.^25,46^ Furthermore, upon heating, some broadening and red shifts are observed for most of the modes, which can at least in part be rationalized by the elongation of chemical bonds.

**Figure 7:**
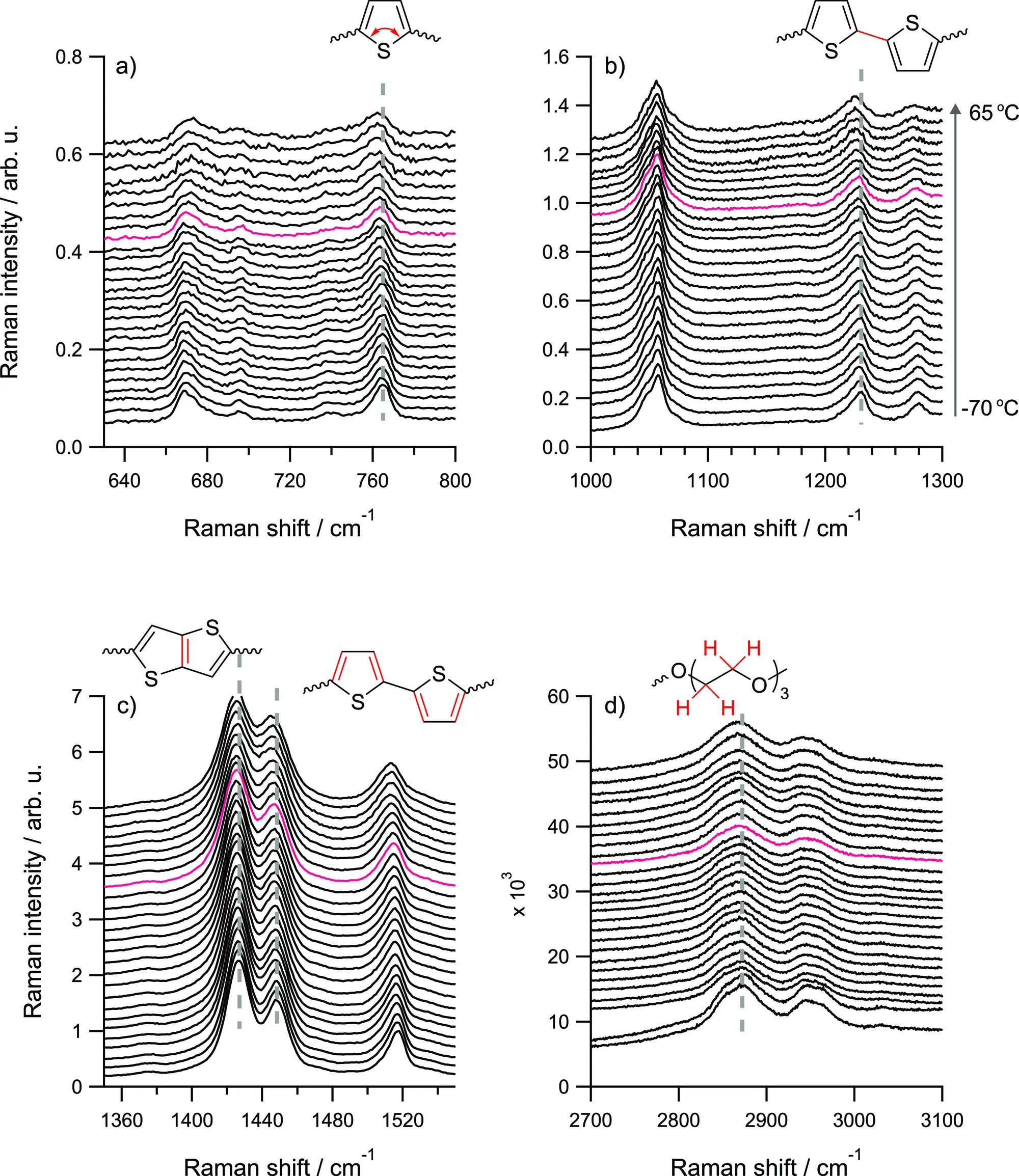
Temperature dependence of the Raman spectrum of p­(g_3_TT-T2), recorded from −70 to 65 °C. Plots (a–d) show the close-up of different spectral regions, where particular vibrational modes appear. The traces in red are those recorded at room temperature.

The temperature dependence of the Raman shift of key vibrational modes, obtained from the peak fitting analysis, reveals variable trends (Figure S11). For instance, deformation modes are less affected by the increasing temperature than the stretching modes. The C–H stretching mode exhibits a red shift upon heating from −70 °C, with a subsequent marked slope change at about −40 °C. This suggests a correlation between the evolution of the Raman shifts and the relaxation of the side chains observed by DMTA (discussed above, Figure 2).

### 3.4 Phenomenological Fits

A red shift arising solely from thermal expansion is expected to follow an approximately linear temperature dependence if no phase transitions occur, due to the smooth increase in average interatomic distances upon heating.^47^ However, across the glass transition, changes in the thermal expansion coefficient lead to deviations from linearity, which can be used to identify transition temperatures. For many of the vibrational modes discussed here (Figure S11), a pronounced nonlinear behavior is observed, suggesting an additional contribution from thermally activated relaxation processes beyond simple thermal expansion. To model these changes, we used the sigmoid function, which is expressed as

$$\omega \left(T\right)=\omega_{0}+\frac{\Delta \omega }{1+e^{(T_{0}-T)/\Delta T}}$$ 4

where ω_0_ is the Raman shift at a temperature just below the transition, Δω is the total Raman shift change across the transition, *T*_0_ is the inflection point corresponding to the midpoint of the transition, *T* is the absolute temperature and Δ*T* defines the characteristic width of the transition (see also the schematic in Figure 8a). The sigmoid function is here used as an empirical model to extract the inflection point *T*_0_. Although it implies symmetric changes around *T*_0_, this is not an assumption for the behavior of the analyzed Raman shifts. Further, a limited range of data points around *T*_0_ is fitted to avoid interference from adjacent relaxation events and hence distortion of the fit. Small variations in the data range fitted did not change the values of *T*_0_ significantly. Notably, this sigmoid model describes the temperature dependence of Raman shifts over both relaxation processes (see Table 2 for the fit parameters).

**Table 2:** Parameters Obtained from Fitting the Thermal Evolution of Raman Shifts with a Sigmoidal Function

vibrational mode	fit parameter	β-relaxation	α-relaxation
ν_s_ C–H (side chain)	ω_0_ (cm^–1^)	2870.5 ± 0.0	
	*T*_0_ (°C)	–54.5 ± 1.4	
	Δω (cm^–1^)	–4.0 ± 0.1	
	Δ*T* (°C)	8.7 ± 1.3	
ν_as_ –C=C–	ω_0_ (cm^–1^)	1516.6 ± 0.2	1514.8 ± 0.1
	*T*_0_ (°C)	–43.5 ± 2.5	33.2 ± 2.6
	Δω (cm^–1^)	–1.9 ± 0.3	–2.4 ± 0.3
	Δ*T* (°C)	10.3 ± 2.5	11.2 ± 2.6
ν_s_ –C=C– (T–T)	ω_0_ (cm^–1^)	1448.9 ± 0.1	1448.0 ± 0.1
	*T*_0_ (°C)	–40.9 ± 1.9	32.2 ± 2.3
	Δω (cm^–1^)	–0.9 ± 0.1	–1.0 ± 0.1
	Δ*T* (°C)	10.2 ± 2.0	10.7 ± 2.3
ν_s_ –C=C– (TT)	ω_0_ (cm^–1^)	1426.5 ± 0.0	1425.3 ± 0.1
	*T*_0_ (°C)	–42.6 ± 1.7	10.2 ± 5.1
	Δω (cm^–1^)	–1.3 ± 0.1	–0.4 ± 0.1
	Δ*T* (°C)	12.9 ± 1.4	5.5 ± 4.2
δ C–S–C	ω_0_ (cm^–1^)	764.4 ± 0.0	763.6 ± 0.2
	*T*_0_ (°C)		37.5 ± 6.1
	Δω (cm^–1^)		–2.0 ± 0.6
	Δ*T* (°C)		9.3 ± 5.2

**Figure 8:**
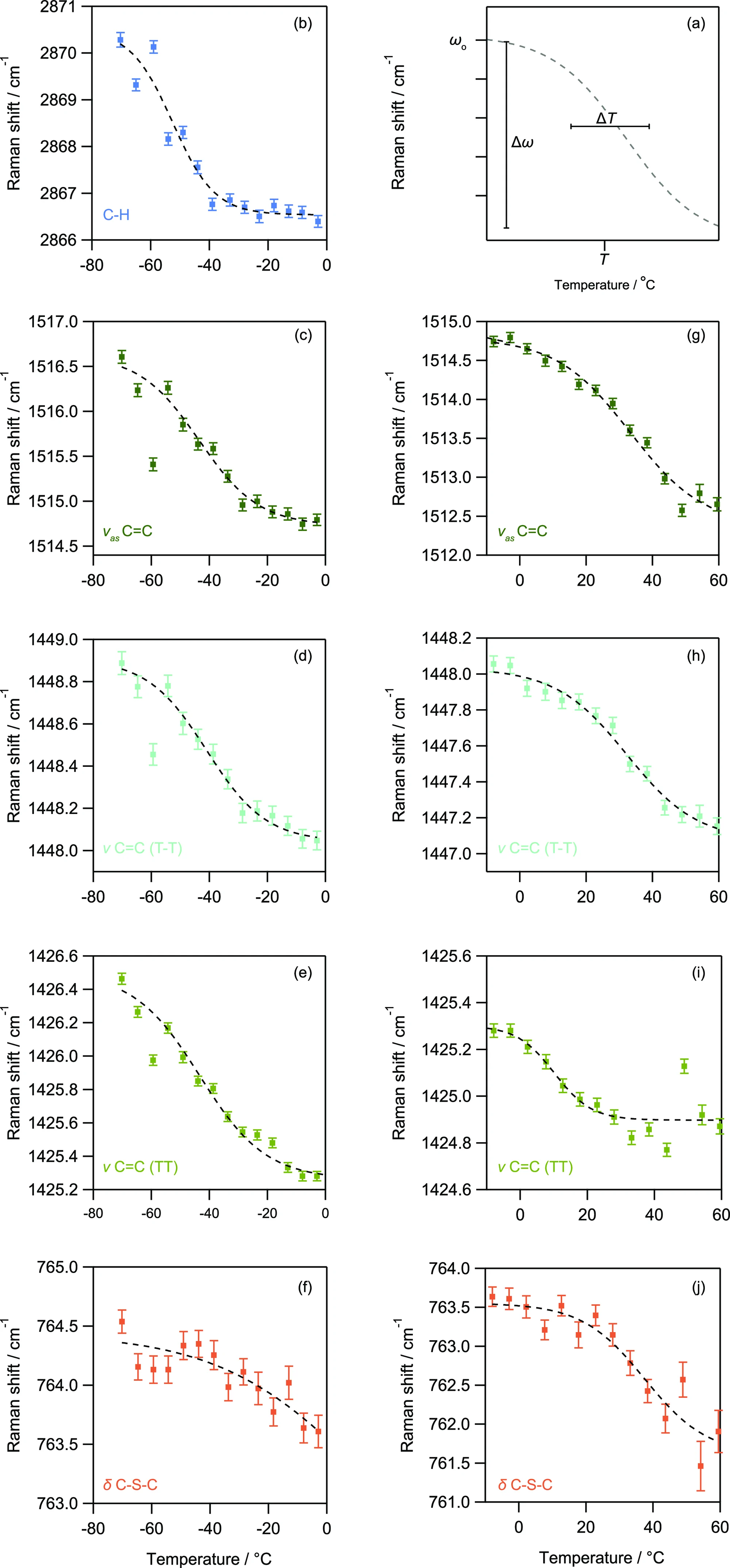
Raman shifts of selected vibrations as a function of temperature. (a) Dashed lines represent their fit by a sigmoid function. (b–f) The temperature region of the β-relaxation, (g–j) the temperature region of the α-relaxation. The data point at −59.4 °C behaves as an outlier and was not included in the fitting with the sigmoid model.

In the temperature region of the β-relaxation, the lowest *T*_0_ is observed for the C–H stretching modes in the side chains (*T*_0_ ∼ −54 °C), followed by the *T*_0_ values of the stretching modes in the rings of the backbone (between −44 and −40 °C). This trend indicates a primary influence of the side chains on the β-relaxation. However, the side-chain mobility also influences the motion of molecular groups in the backbone, for instance the Raman-active −C=C– stretching modes that are part of the effective conjugation coordinate (ECC) and hence sensitive to conjugation length and bond alternation. The transition temperatures identified by Raman spectroscopy are consistent with those of the relaxation captured by FSC (Figure S6) and are further supported by DMTA (Figure 2d), wherein the relaxation follows an Arrhenius trend, characteristic of sub-*T*_g_ relaxation processes. These observations support the hypothesis that the lowest-temperature phenomenon is primarily associated with the relaxation of the side chains,^9^ corresponding to localized motions of small molecular units^48^ that in turn may also influence the vibrations of nearby molecular groups. Notably, in this low-temperature range, the Raman shifts of the inter-ring C–C stretch, C–H bending, and C–S–C bending exhibit a much weaker temperature dependence (see Figure S11).

In the temperature range of the α-relaxation, the sigmoid fits reveal that the C–S–C bending mode, as well as the −C=C– (ν_as_) and −C=C– (T2) stretching modes, have a *T*_0_ value in the range of 32–37 °C. This is preceded by a lower *T*_0_ value at ∼10 °C for the −C=C– (TT) mode. The onset of backbone relaxation in this higher-temperature range is consistent with the mechanical relaxation observed by DMTA. The involvement of vibrational modes associated with the polymer backbone suggests that this relaxation process is governed by cooperative segmental motion.^49^ This correlation supports the hypothesis that the polymer backbone plays a central role in *T*_g_. In particular, the sensitivity of the C–S–C bending mode in this temperature region may reflect changes in backbone conformation, as this vibration is directly coupled to torsional and angular fluctuations along the chain. Further, with *T*_0_ ranging from 10 to 37 °C rather than taking a single value, Raman spectroscopy reveals a sequence of local dynamics, possibly reflecting the broadness of the α-relaxation shown in Figure 2a.

### 3.5 Correlation between Energy Dissipation and Raman Shifts

A direct comparison of the temperature dependence of the loss modulus (*E*″, dashed red line) and of the Raman shift of ν_s_ −C=C– (TT, blue dots) shows a correlation between mechanical and vibrational properties (Figure 9). In fact, despite originating from two independent and inherently different experimental techniques, the two data sets exhibit remarkably similar trends. More precisely, both the Raman shift and the *E*″ modulus decrease with increasing temperature, showing inflection points at very proximate temperatures.

**Figure 9:**
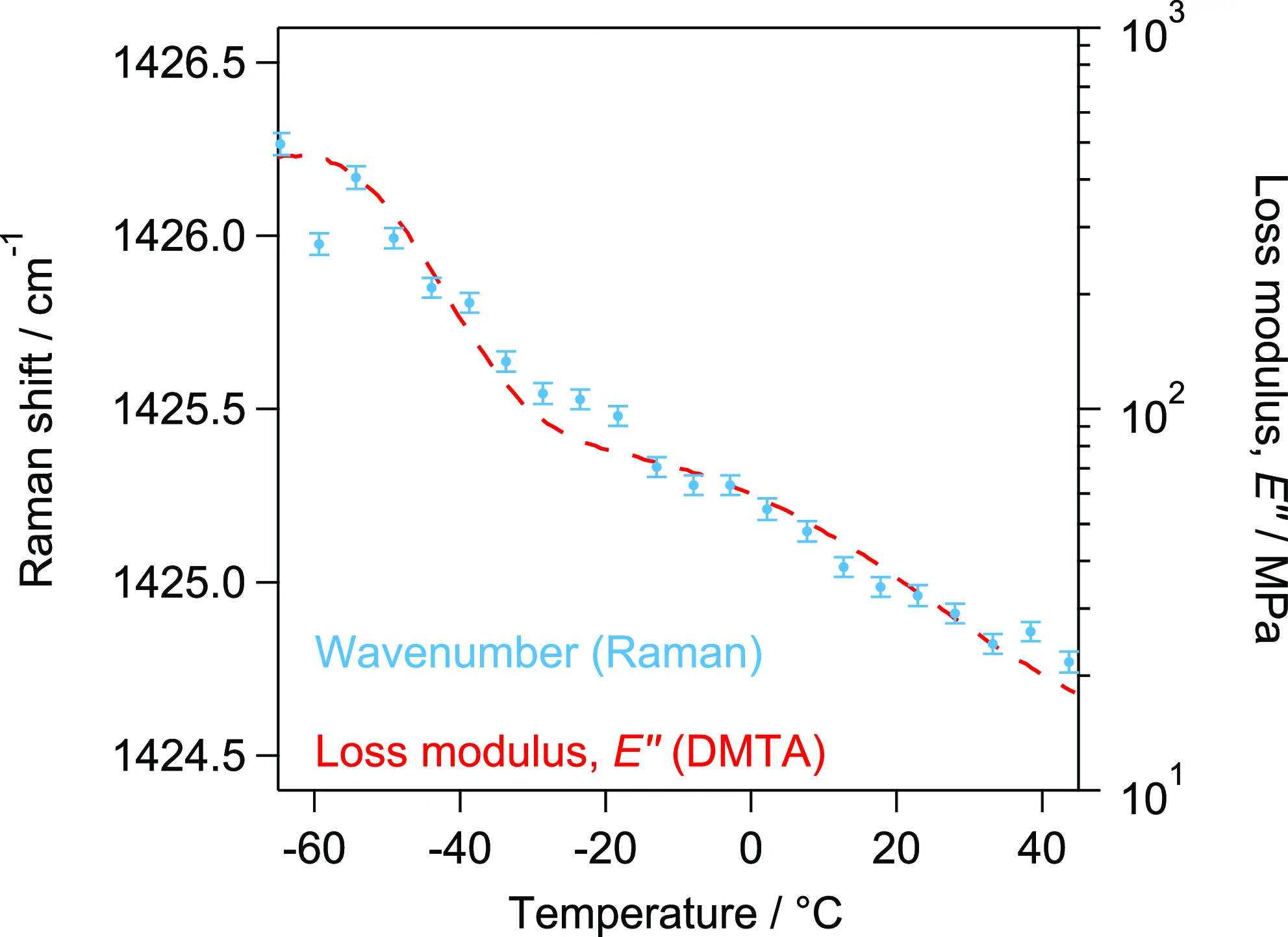
Temperature dependence of the Raman shift (left axis, blue dots, temperature scanned at 20 °C/min) and of the loss modulus (*E*″, right axis, dashed red line, measured at 1 Hz and temperature scanned at 15 °C/min), independently measured for p­(g_3_TT-T2).

Such a correlation is not entirely unexpected, since the dissipation of energy, in the case of DMTA expressed as *E*″, can affect the rate of change of the Raman shifts, which is exactly what is probed by the temperature-dependent spectroscopic measurements. Hence, the evolution of Raman shifts serves as a window to the molecular dynamics and conformational changes that govern energy dissipation within a material. Despite the remarkable agreement shown in Figure 9, it is important to note that this situation may slightly change if the comparison is made to DMTA data recorded at other frequencies or scan rates. Indeed, we emphasize that it is the fluctuation with temperature that agrees between *E*″ and Raman shifts, rather than the exact matching values. It is also worth commenting on the effects that heating rates and film confinement can have on the apparent *T*_g_ values, with faster heating rates tending to increase *T*_g_ while thinner films can feature either lower or higher *T*_g_ values.

Although correlative Raman–mechanical studies have been applied to inorganic materials before, such as glasses, ceramics, and carbon-based materials, many times these studies have been conducted at room temperature and aimed at demonstrating that Raman shifts can be used to estimate *E*′.^50,51^ On the other hand, temperature-dependent Raman spectroscopy has been used sporadically to detect relaxations in polymers, but typically through the detection of discontinuous changes in Raman shifts or intensities in commodity polymers such as polyamides and polystyrene, without a deeper phenomenological analysis.^25,52^ In the field of conjugated polymers, glass transitions and mechanical properties (and possibly their correlation) have typically been studied by calorimetric and mechanical methods. Hence, to the best of our knowledge, our study is the first to correlate mechanical and vibrational properties, demonstrating that Raman spectroscopy is able to capture the underlying molecular-scale dynamical changes during structural relaxation.

## 4 Conclusions

In conclusion, we show that DMTA and Raman spectroscopy provide complementary insights into molecular relaxations across macroscopic and molecular scales. We present an experimental approach to assigning vibrational modes by studying monomeric units and individual building blocks, which are compared with the spectra of the polymer. Notably, this is the first time that this combined methodology has been applied to a conjugated polymer to probe relaxation processes, including the glass transition.

In particular, the combination of these two techniques enables the identification of both phenomena, the β- and α-relaxations, and the specific chemical bonds associated with each process. Our results show that the β-relaxation is accompanied by the softening of the C–H stretching modes in the side chains, while the α-relaxation shows a strong correlation with Raman shift changes of vibrational modes in the polymer backbone.

In future studies, it will be interesting to use this temperature-dependent Raman spectroscopy approach to elucidate the molecular origin of relaxation processes in doped conjugated polymer films. In all cases, and upon change of the polymer, the ground must be a careful and correct assignment of the analyzed vibrational modes. In more general terms, vibrational spectroscopy can be correlated with mechanical properties of materials, such as *E*′ and *E*″, by being sensitive to both covalent bonds and intermolecular interactions.

## Supplementary Material



## Notes

Additional experimental details, structures, and information on small molecules and polymers investigated; thin-film image; thermal characterization including TGA, DMTA, and FDSC; Raman peak fitting methodology; supporting Raman spectra of polymer backbones and side chains; examples of quantitative Raman analysis; and temperature-dependent Raman contour plots, and Raman peak shift analysis as a function of temperature (PDF)
