# Supplementary material for: Linking the Evolution of Raman-Active Vibrational Modes to the Glass Transition of a Polymer Mixed Conductor
Source: Chem Mater. 2026 Aug 29;38(18):9361–72. doi: 10.1021/acs.chemmater.6c01379 (PMC13603066; doi:10.1021/acs.chemmater.6c01379)
Supplement: Supplementary file 1 [file cm6c01379_si_001.pdf]

Supplementary Information

Linking the evolution of Raman active vibrational modes to the glass transition of a polymer mixed conductor

Giannis G. Gkikas, Elsa Veronica Flores-Vela, Mariavittoria Craighero, Joost Kimpel, Christian Müller, and Anna Martinelli*

Department of Chemistry and Chemical Engineering, Chalmers University of Technology, Gothenburg 41296, Sweden

E-mail: anna.martinelli@chalmers.se

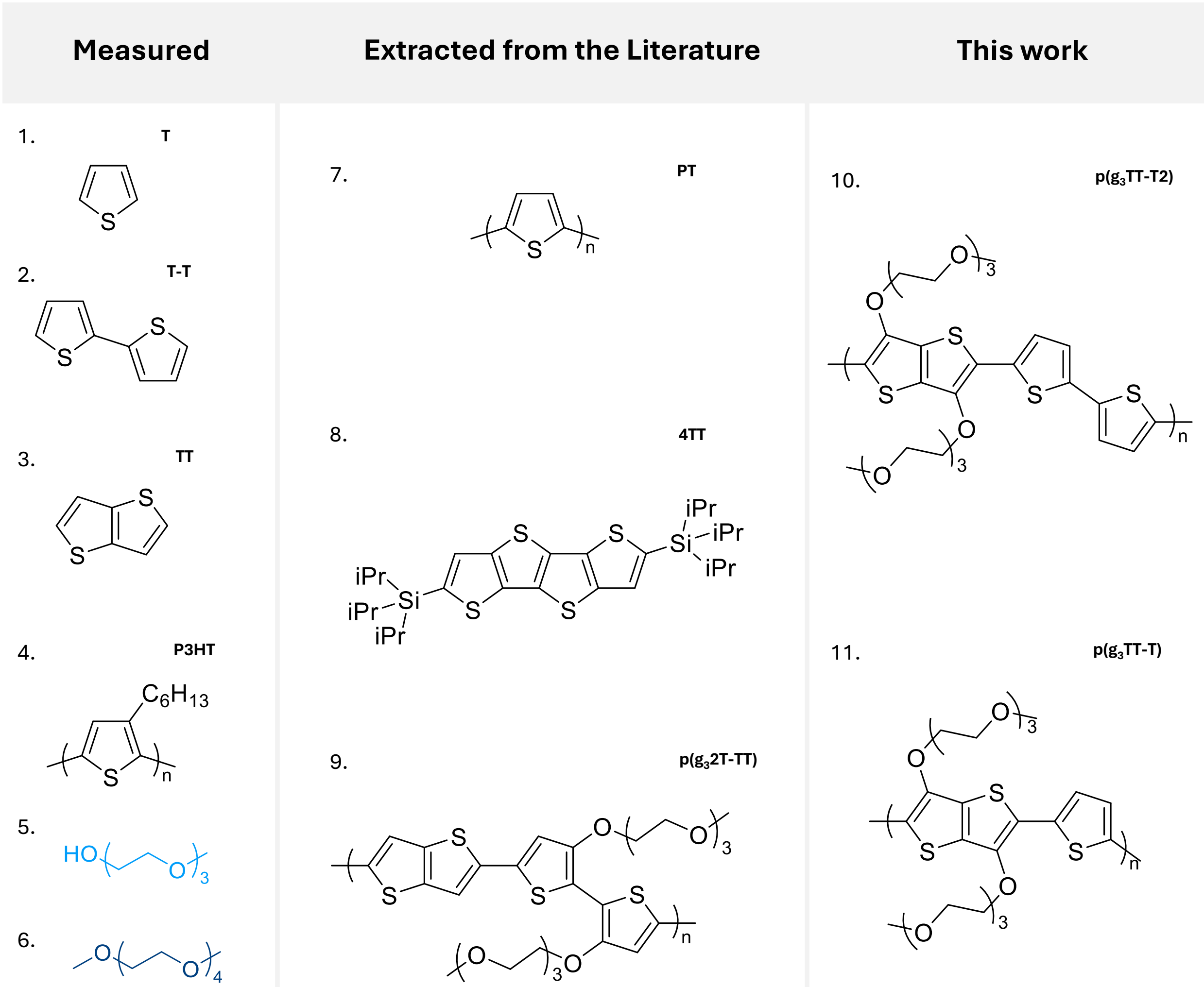

Figure S1: Small molecules and polymers investigated in this work and used to help in the assignment of vibrational modes observed in the Raman spectrum of p(g_3TT-T2). The Raman spectra of samples 1-6 and 10-11 were measured, while the Raman spectra of samples 7-9 were taken from the literature.

.

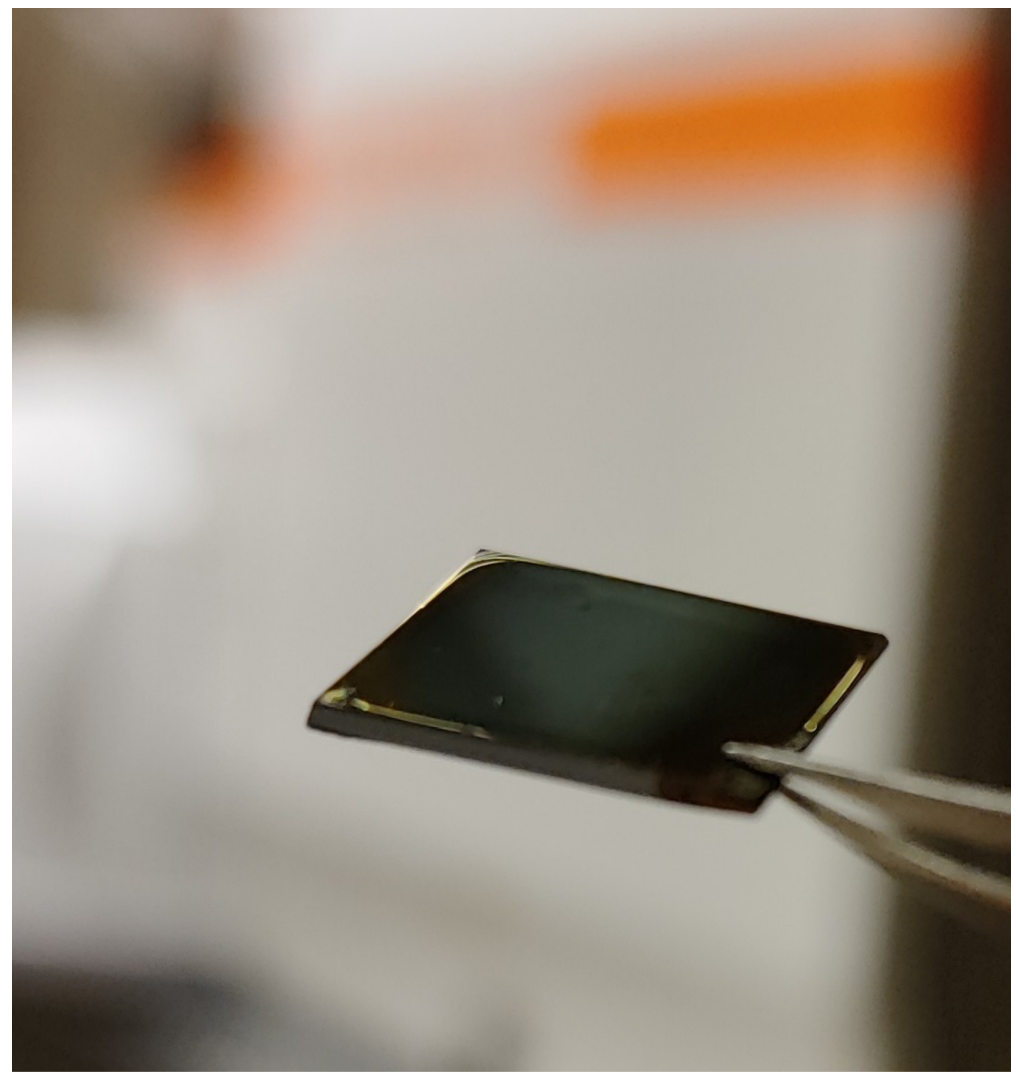

Figure S2: Image of a thin film of p(g_3TT-T2) deposited onto a substrate of Si. The blurred, white and orange background is the housing of an InVia-Reflex Raman spectrometer.

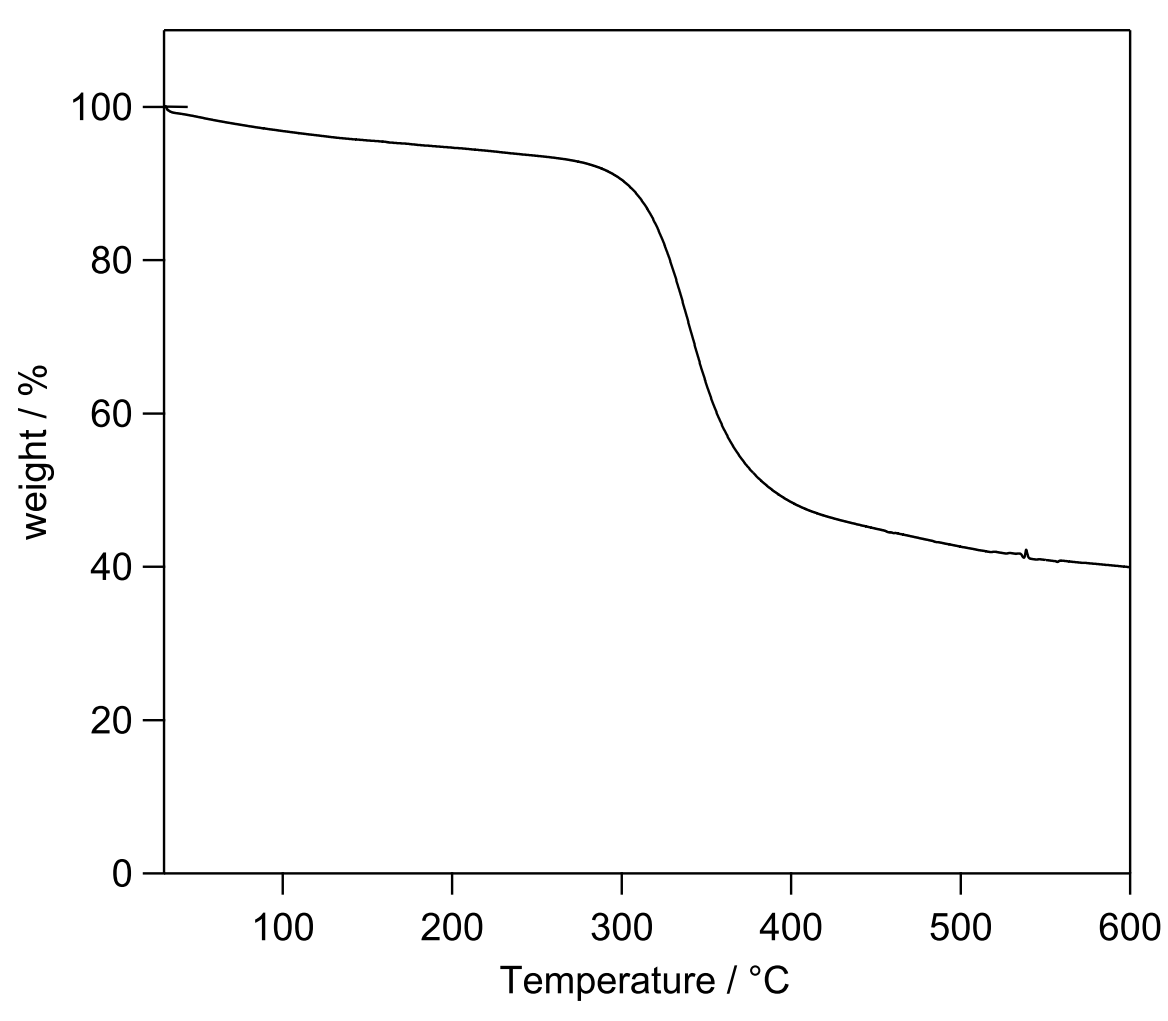

Figure S3: Thermogravimetric analysis (TGA) thermogram recorded for p(g_3TT-T2) at a heating rate of $10\,^{\circ}\mathrm{C\,min^{-1}}$.

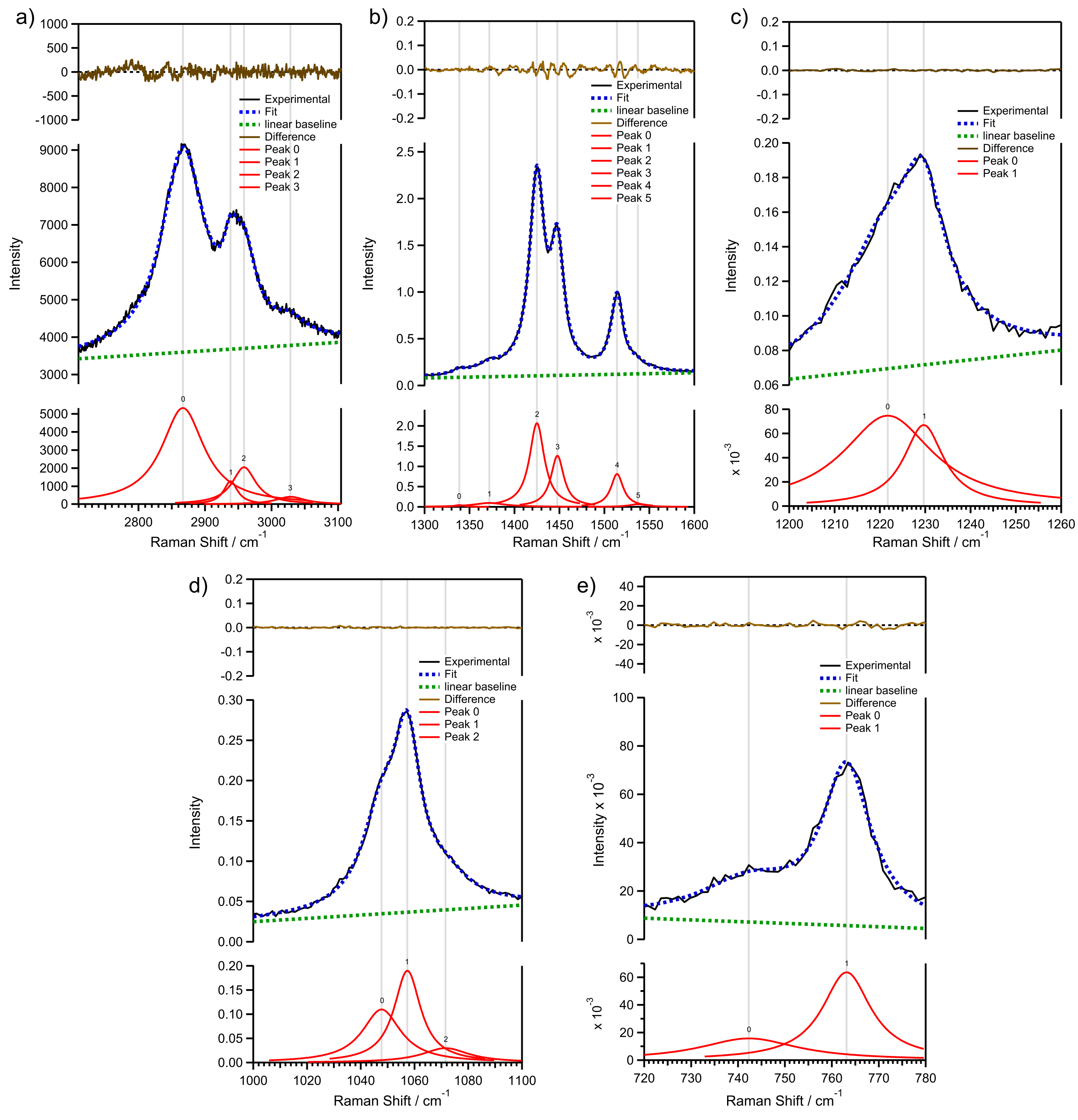

Figure S4: Peak fitting in the spectral regions: a) 2700–3100 cm^{-1}, b) 1300–1600 cm^{-1}, c) 1200–1260 cm^{-1}, d) 1000–1100 cm^{-1}, and e) 720–780 cm^{-1} of the Raman spectrum recorded at room temperature from a thin film of p(g_3TT-T2).

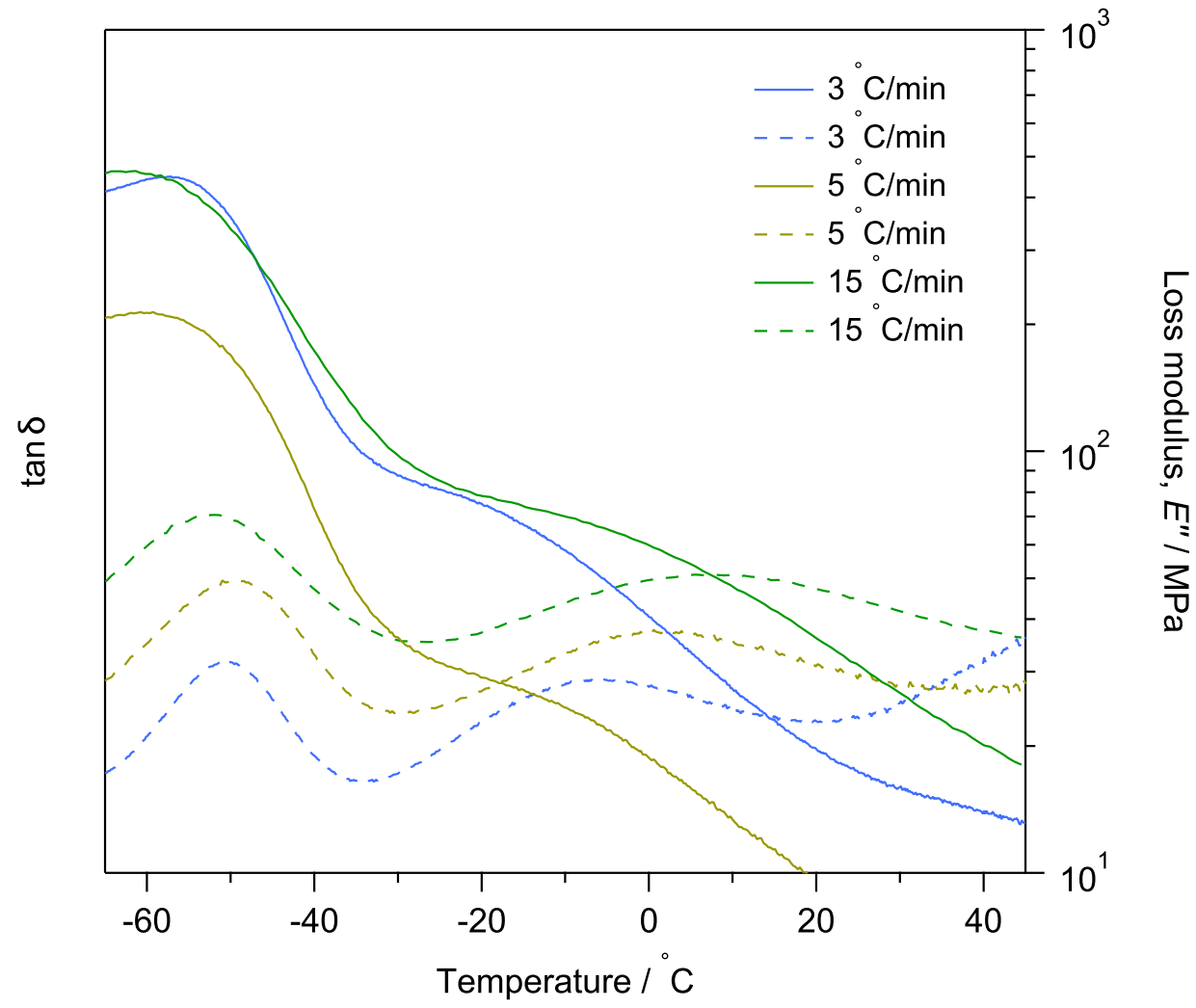

Figure S5: DMTA curves recorded for p(g$_3$TT-T2) at different scan rates, i.e. 3, 5 and 15 °C/min. The curves for $\tan\delta$ are shown as dashed lines, while those for loss modulus (E'') are shown as solid lines.

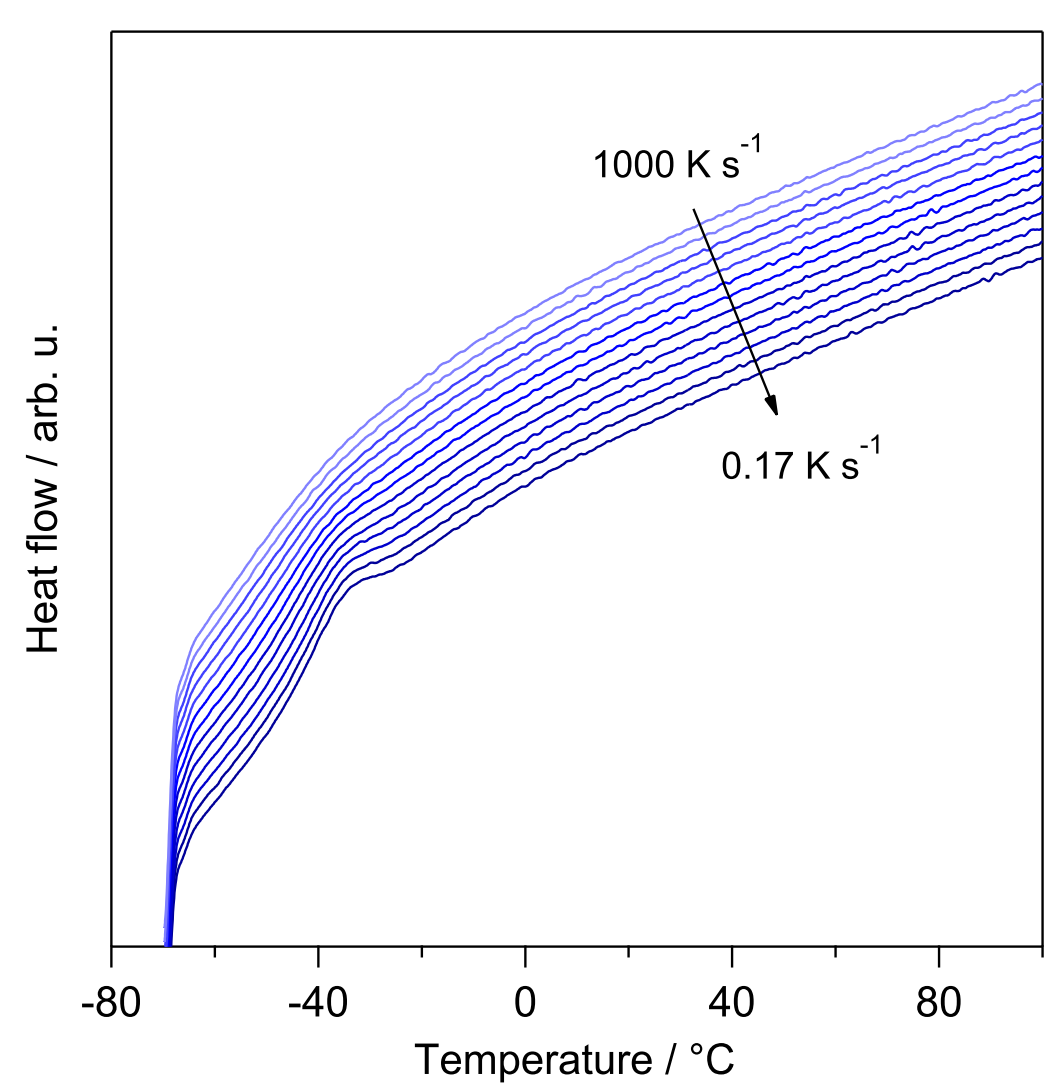

Figure S6: Fast scanning calorimetry (FSC) thermograms recorded for p(g$_3$TT-T2) at different cooling rates, from $1000\,\mathrm{K\,s^{-1}}$ to $0.17\,\mathrm{K\,s^{-1}}$.

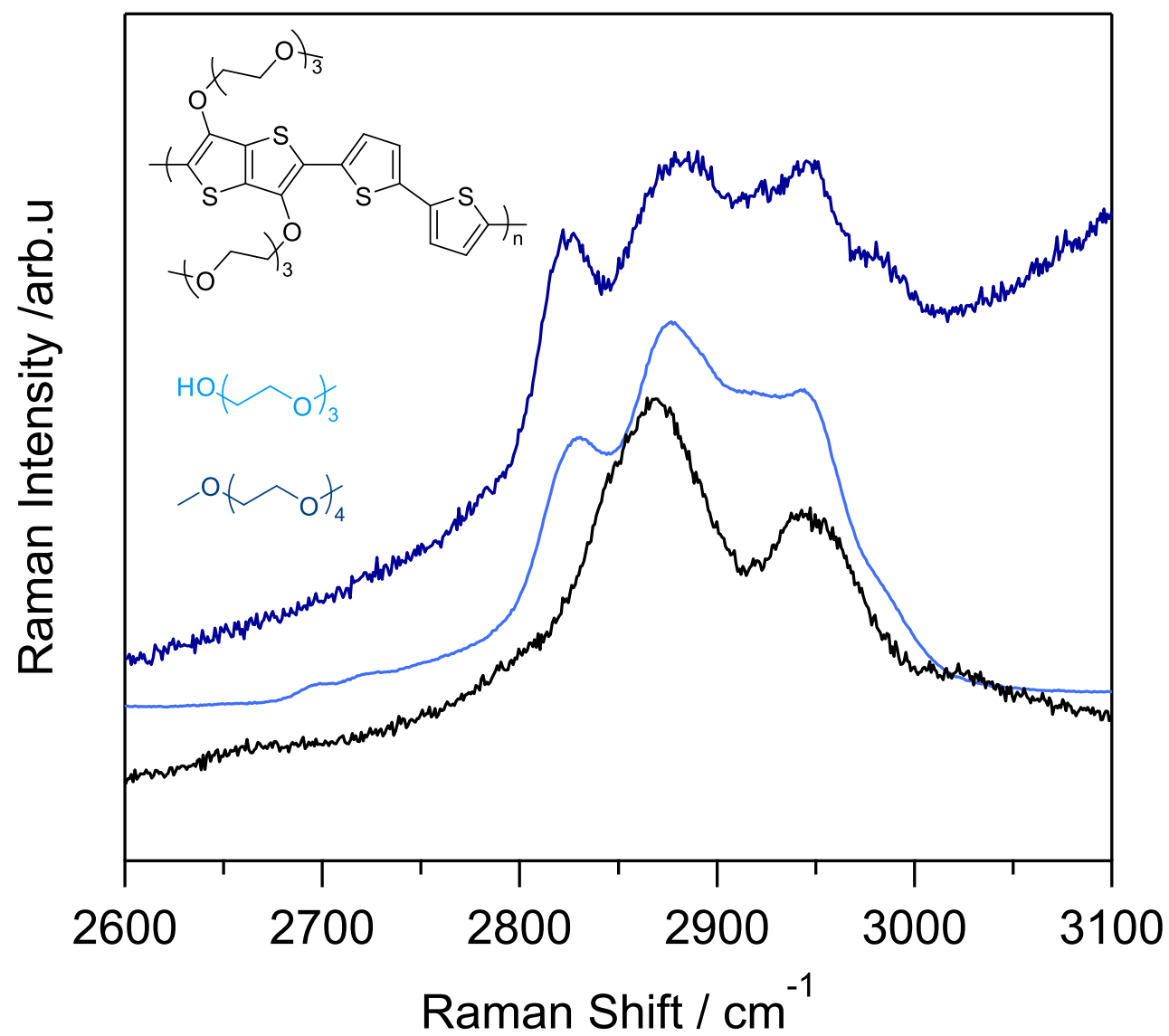

Figure S7: Raman spectra at room temperature of p(g_3TT-T2) (black), triethylene glycol monomethyl ether (royal blue) and tetraethylene glycol dimethyl ether (navy blue), in the spectral range of CH stretching modes.

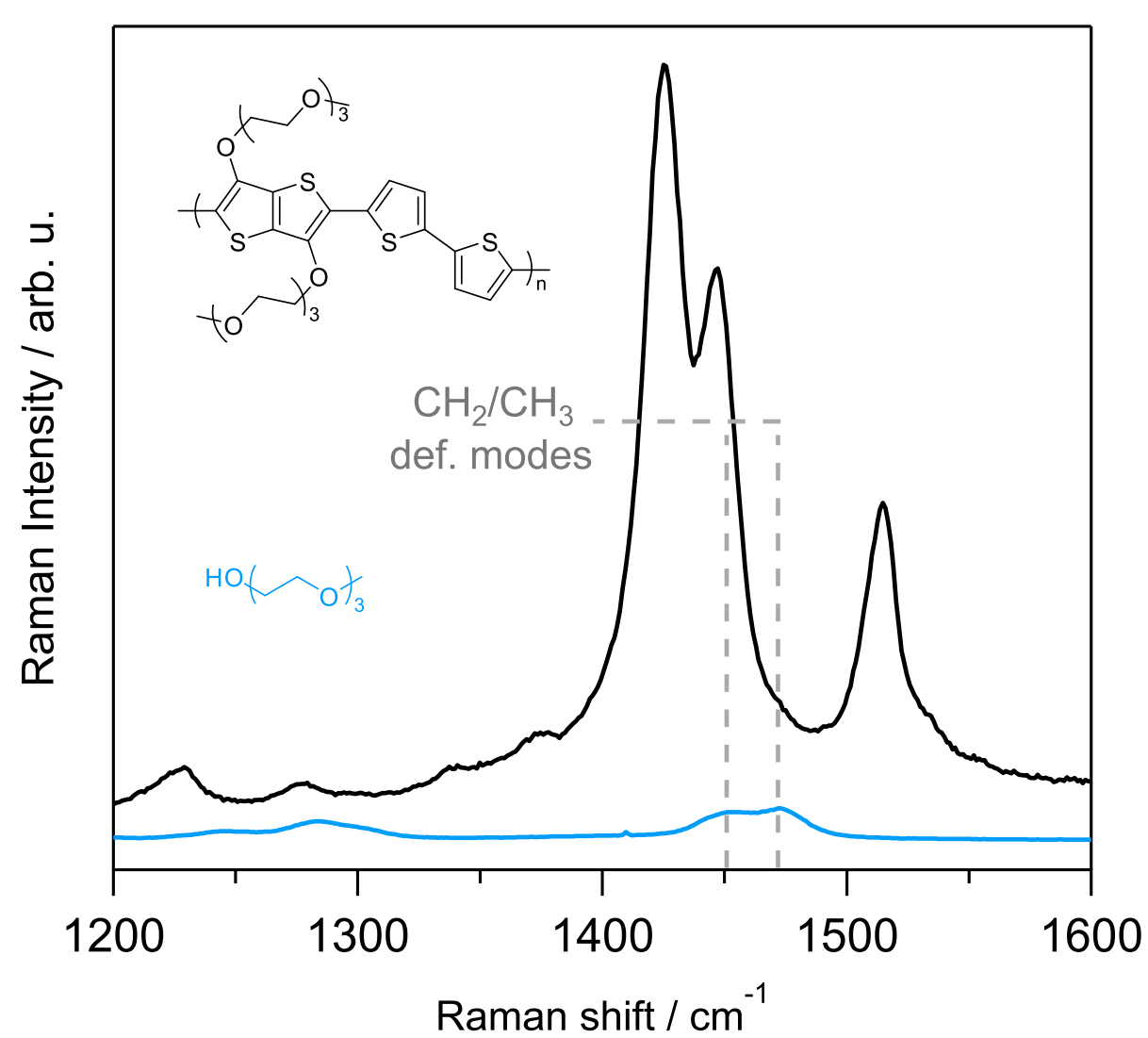

Figure S8: Comparison of the Raman spectra recorded for p(g_3TT-T2) and triethylene glycol monomethyl ether (royal blue).

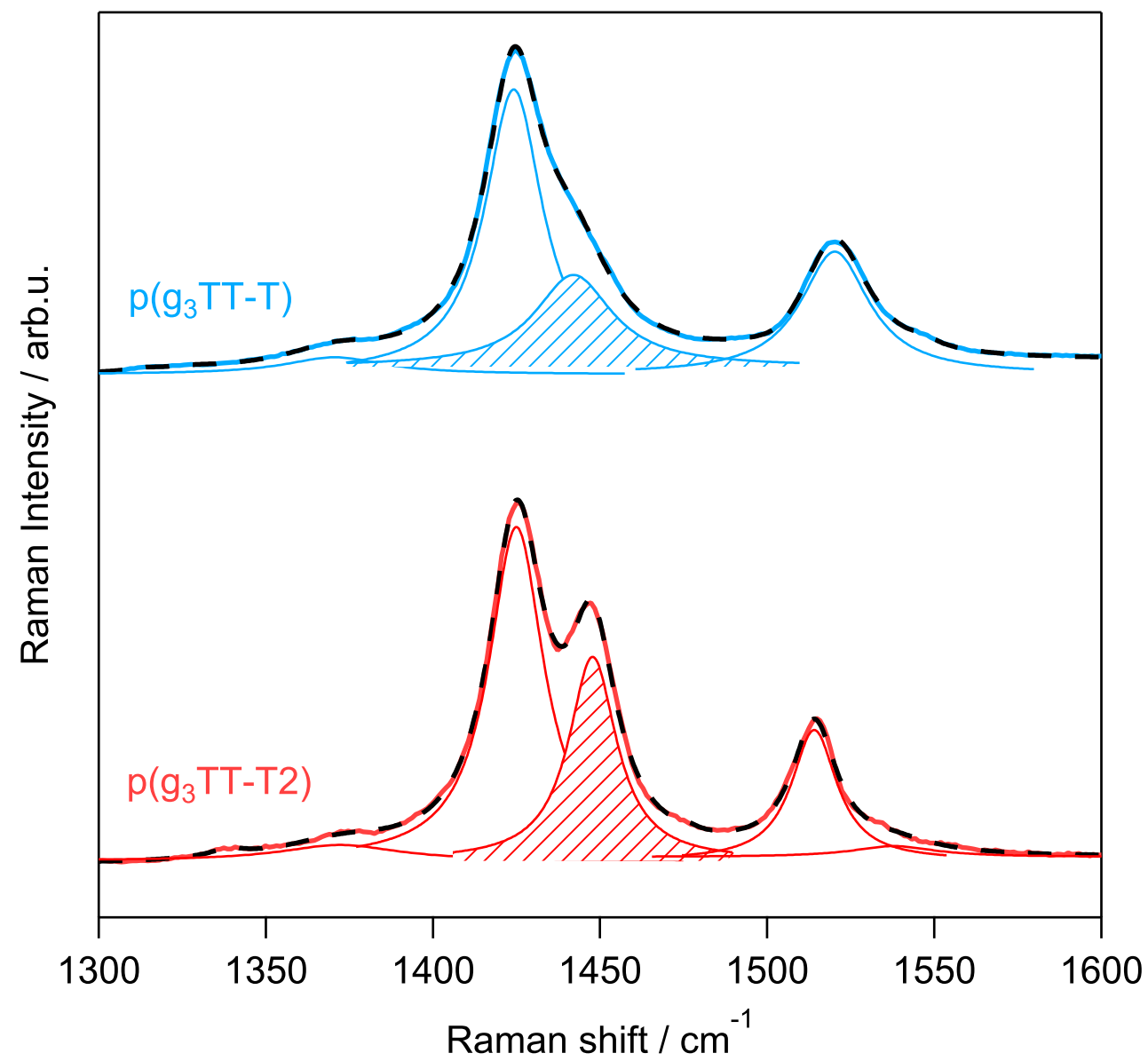

Figure S9: Quantitative analysis, in the region of the -C=C- stretching modes, of the Raman spectra of p(g_3TT-T) (blue) and p(g_3TT-T2) (red). Peak fitting reveals that the dashed area relative the area under the peak at 1425 cm^{-1} is 0.38 in p(g_3TT-T) and 0.50 in p(g_3TT-T2), reflecting the relative number of repeating T units in each molecular structure.

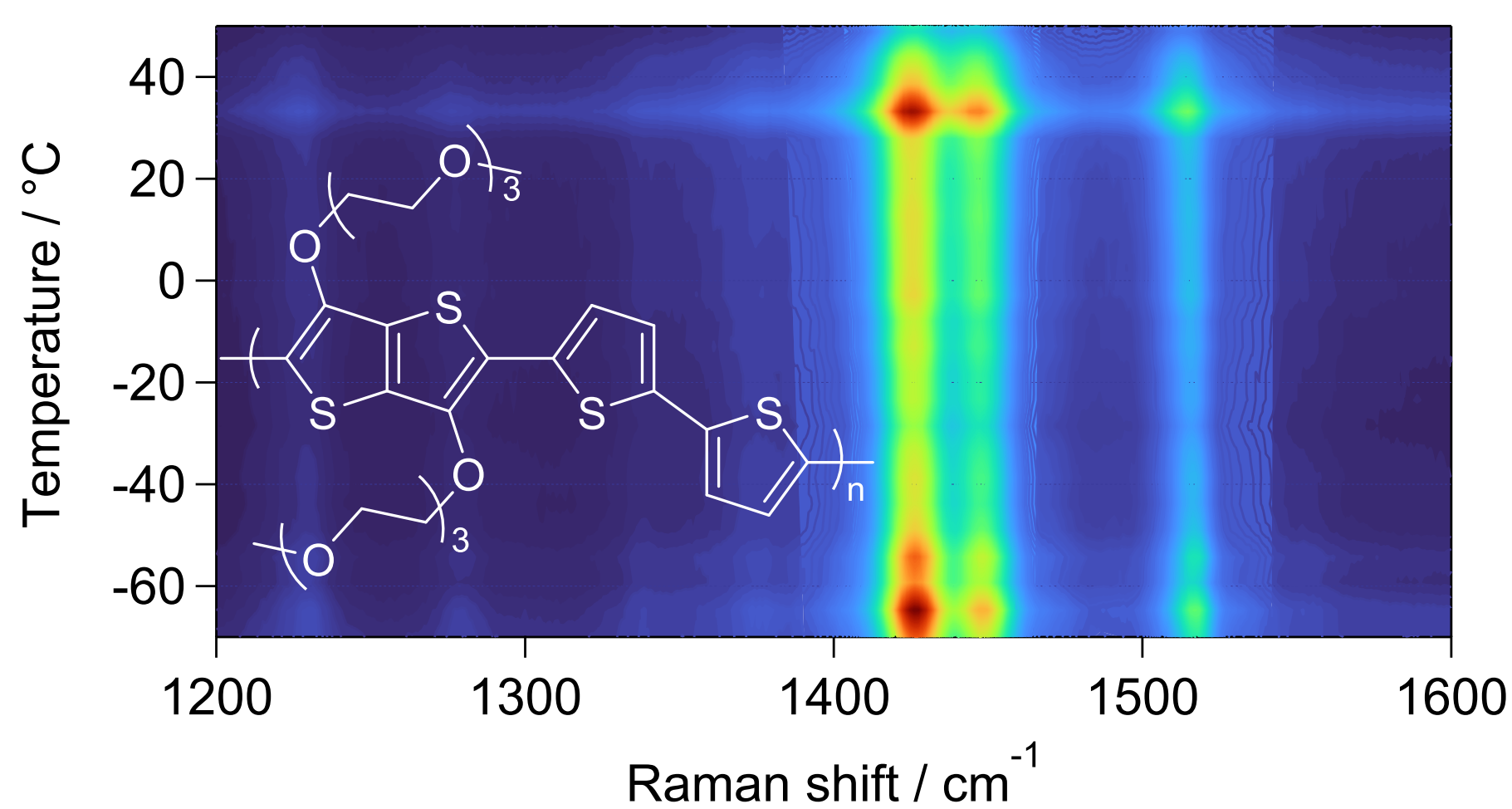

Figure S10: Contour plot of the p(g_3TT-T2) Raman spectra recorded during heating, showing the evolution of Raman intensity as a function of temperature (red=higher intensity, blue=lower intensity). This contour plot was created from raw, not-normalized Raman spectra.

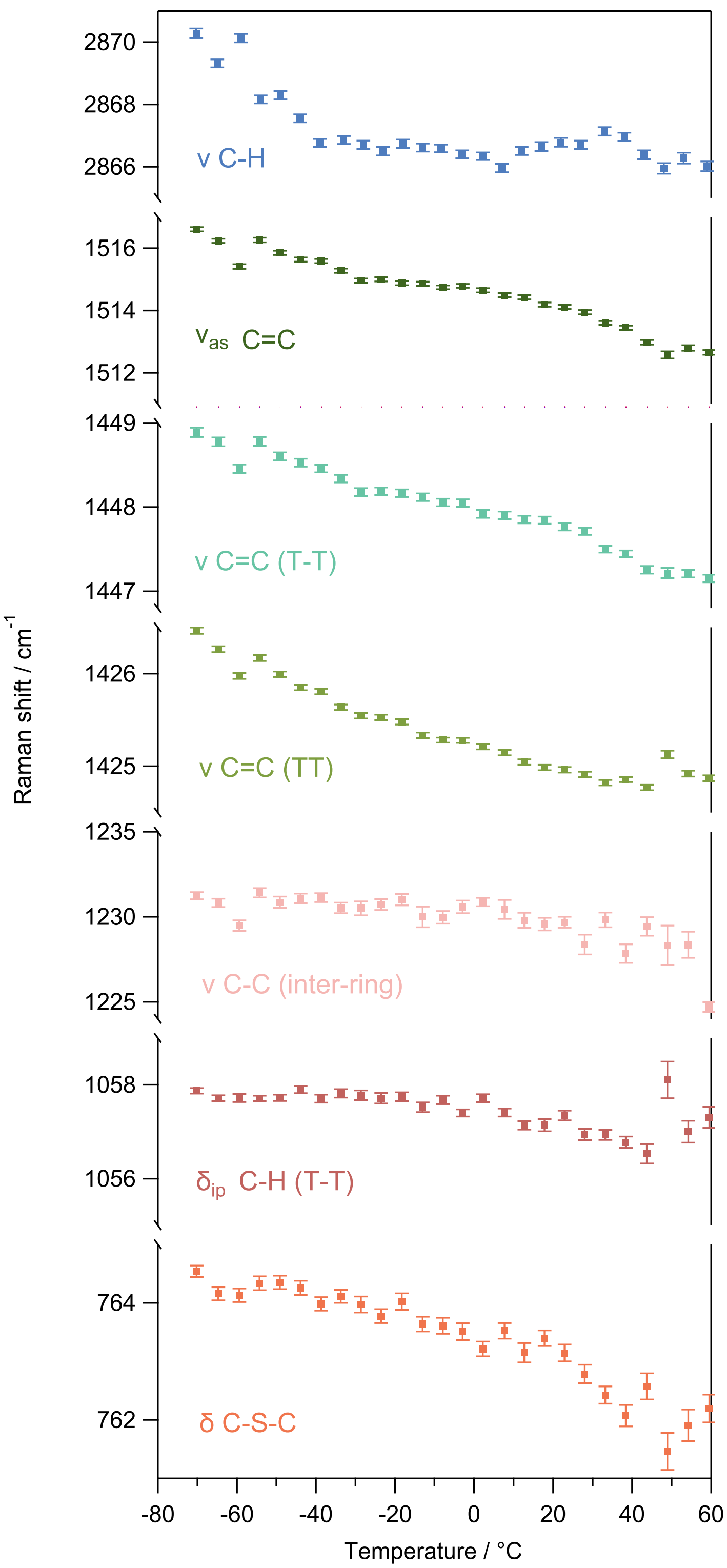

Figure S11: Temperature dependence of the Raman shift of selected vibrational modes, as obtained from the peak fitting analysis. The error bars shown are from the fitting procedure.
